# Supplementary material for: Processed foods purchase profiles in urban India in 2013 and 2016: a cluster and multivariate analysis
Source: BMJ Open. 2022 Oct 7;12(10):e062254. doi: 10.1136/bmjopen-2022-062254 (PMC9558783; doi:10.1136/bmjopen-2022-062254)

**Supplementary Appendix**

*Supplementary Table 1: Food Groups*

| | Food group | Individual food items |
|---|---|---|
| 1 | Staples | Basmati rice [a], atta |
| 2 | Milk | Milk |
| 3 | Oils | Edible oil, ghee, vanaspati |
| 4 | Salt | Salt |
| 5 | Processed Wheat | Pasta, bread |
| 6 | Tea and Coffee | Tea, coffee |
| 7 | Spices | Spices |
| 8 | Butters and Cheese | Butter, cheese |
| 9 | Salty Snacks (UPF) | Chips, collet, popcorn, potato chips, puffed snacks, traditional snacks |
| 10 | Drinks (UPF) | Carbonated drinks, juices, milk based drink, squash |
| 11 | Ready to Eat Foods (UPF) | Cooking paste, cook mix, noodle, soup, sauces, pickles, ready meals |
| 12 | Sweet Snacks (UPF) | Biscuits, rusk, chocolate, chocolate spread, peanut, butter, jams |
| 13 | Milk Drinks (UPF) | Milk food drinks (e.g. malt drinks, chocolate milk), Milk powder (including infant milk powder) |
| 14 | Frozen Foods (UPF) | Frozen foods (e.g. fish fingers, samosas) |
| 15 | Breakfast cereals (UPF) | Breakfast cereals |

Note: [a] Includes data on basmati rice only, which is a higher quality rice and thus does not include the non-basmati variety of rice that is consumed by most Indians

*Supplementary Table 2: Calinski and Harabasz pseudo-F index*

| Clusters | 2013 | 2016 |
|---|---|---|
| 3 | 37,991.23 | 41,112.57 |
| 4 | 36,085.22 | 37,939.35 |
| 5 | 37,141.41 | 37,687.36 |
| 6 | 35,393.65 | 36,583.87 |
| 7 | 36,005.66 | 35,706.64 |
| 8 | 34,889.70 | 34,865.53 |

*Supplementary Table 3: Cluster multinomial logit regressions*

| | Cluster | |
|---|---|---|
| VARIABLES | 2013 | 2016 |
| Base Cluster Low | | |
| Cluster - Medium | | |
| SES- Middle Class | 1.388*** (0.102) | 1.548*** (0.097) |
| SES- Upper Middle Class | 2.133*** (0.247) | 2.243*** (0.23) |
| SES- Upper Class | 3.526*** (0.531) | 3.792*** (0.426) |
| Town Population - 500k>=X>100k | 1.048 (0.161) | 0.979 (0.147) |
| Town Population - 1mil>=X>500k | 0.934 (0.159) | 1.008 (0.172) |
| Town Population - 4mil>=X>1mil | 1.041 (0.191) | 0.973 (0.167) |

| | | |
|---|---|---|
| Town Population - >4mil | 0.835 (0.217) | 0.756 (0.173) |
| Household Size | 0.542*** (0.017) | 0.564*** (0.022) |
| Infant | 1.195* (0.089) | 1.089 (0.098) |
| Children under 1 year | 1.001 (0.067) | 1.042 (0.099) |
| Children 2-4 years | 1.044 (0.052) | 0.996 (0.039) |
| Children 5-9 years | 0.883*** (0.029) | 0.898* (0.04) |
| Children 10-14 years | 0.836*** (0.027) | 0.837*** (0.023) |
| Children 15-17 years | 0.706*** (0.028) | 0.805*** (0.024) |
| Durable: Colour TV | 1.17* (0.086) | 1.5*** (0.137) |
| Durable: Refrigerator | 1.534*** (0.074) | 1.358*** (0.074) |
| Durable: Washing Machine | 1.45*** (0.1) | 1.412*** (0.115) |
| Durable: Laptop/PC | 1.271*** (0.069) | 1.171** (0.058) |
| Durable: Four Wheeler | 1.483** (0.179) | 1.288** (0.122) |
| Durable: AC | 1.846*** (0.216) | 1.816*** (0.201) |
| Constant | 5.826*** (1.307) | 7.4*** (1.767) |
| | | |
| Cluster - High | | |
| SES- Middle Class | 1.546* (0.283) | 1.665*** (0.214) |
| SES- Upper Middle Class | 3.278*** (0.691) | 3.64*** (0.617) |
| SES- Upper Class | 5.87*** (1.507) | 9.556*** (1.95) |
| Town Population - 500k>=X>100k | 1.35 (0.473) | 0.808 (0.193) |
| Town Population - 1mil>=X>500k | 0.629 (0.23) | 0.718 (0.181) |
| Town Population - 4mil>=X>1mil | 1.143 (0.321) | 0.742 (0.201) |
| Town Population - >4mil | 0.892 (0.418) | 0.436* (0.181) |
| Household Size | 0.157*** (0.025) | 0.174*** (0.018) |
| Infant | 0.923 (0.297) | 1.289 (0.229) |
| Children under 1 year | 1.403 (0.373) | 0.996 (0.308) |
| Children 2-4 years | 1.2 (0.255) | 1.104 (0.168) |
| Children 5-9 years | 0.747** (0.072) | 0.981 (0.085) |
| Children 10-14 years | 0.804 (0.103) | 0.734** (0.067) |
| Children 15-17 years | 0.486*** (0.054) | 0.731*** (0.051) |
| Durable: Color TV | 1.668*** (0.23) | 2.714*** (0.476) |
| Durable: Refrigerator | 1.435** (0.17) | 1.428** (0.16) |
| Durable: Washing Machine | 1.597** (0.215) | 1.681*** (0.194) |
| Durable: Laptop/PC | 1.478** (0.185) | 1.335** (0.113) |
| Durable: Four Wheeler | 2.208*** (0.439) | 1.916*** (0.26) |
| Durable: AC | 3.199*** (0.844) | 3.433*** (0.552) |
| Constant | 15.488*** (7.106) | 38.817*** (17.155) |
| | | |
| Observations | 58,878 | 58,878 |
| State Effect | Yes | Yes |

Notes: Robust standard errors in parentheses; *** p<0.001, ** p<0.01, * p<0.5

*Supplementary Figure 1: Distribution of Processed Food Group Diversity Score in 2016*

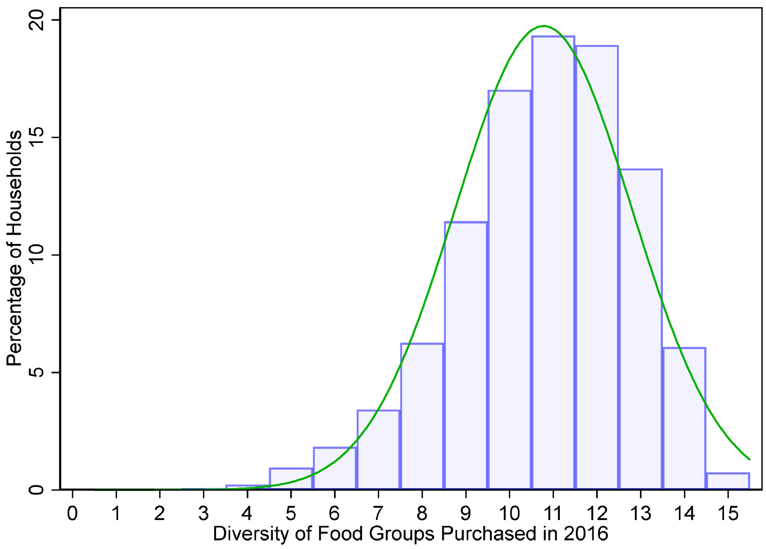


*Supplementary Figure 2: Distribution of annual Ultra-Processed food and Beverages Quantity in 2016*

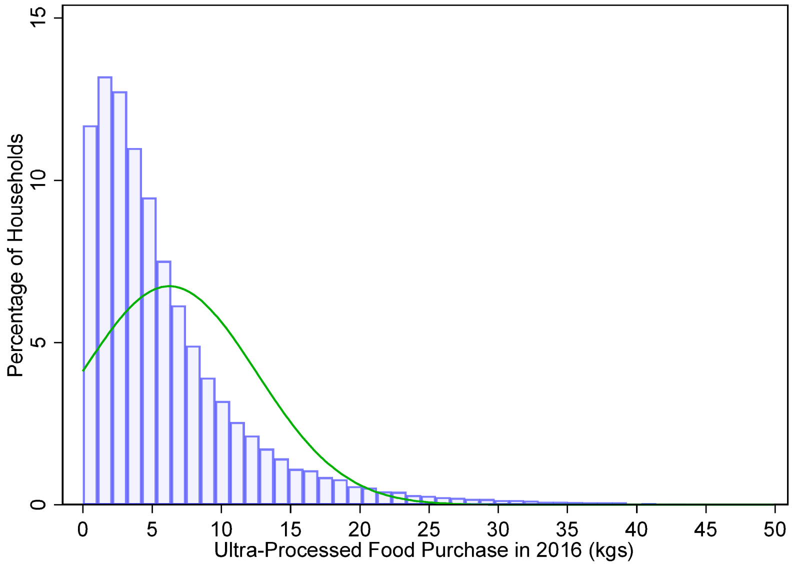


Notes: For readability purposes, the distribution excludes 140 households (0.2%) that purchased more than 50kgs of UPF per household member.

*Supplementary Figure 3: Kernel Density Curves for Processed Food Purchase by Socio-Economic Status in 2016*

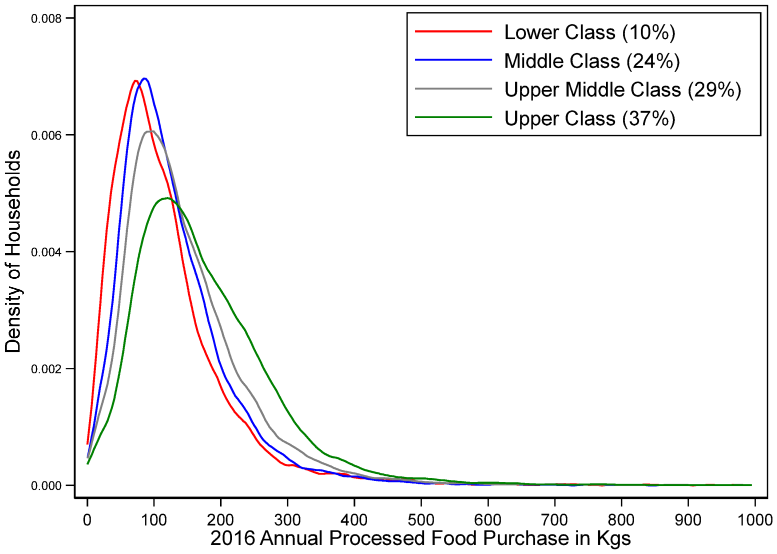

Supplement: Supplementary data [file bmjopen-2022-062254supp001.pdf]
