# Supplementary material for: Processed foods purchase profiles in urban India in 2013 and 2016: a cluster and multivariate analysis
Source: BMJ Open. 2022 Oct 7;12(10):e062254. doi: 10.1136/bmjopen-2022-062254 (PMC9558783; doi:10.1136/bmjopen-2022-062254)

BMJ Open is committed to open peer review. As part of this commitment we make the peer review history of every article we publish publicly available.

When an article is published we post the peer reviewers' comments and the authors' responses online. We also post the versions of the paper that were used during peer review. These are the versions that the peer review comments apply to.

The versions of the paper that follow are the versions that were submitted during the peer review process. They are not the versions of record or the final published versions. They should not be cited or distributed as the published version of this manuscript.

BMJ Open is an open access journal and the full, final, typeset and author-corrected version of record of the manuscript is available on our site with no access controls, subscription charges or pay-per-view fees (http://bmjopen.bmj.com).

If you have any questions on BMJ Open's open peer review process please email info.bmjopen@bmj.com

BMJ Open

Longitudinal analysis of processed foods purchase profiles for urban Indian households

Journal:	*BMJ Open*
Manuscript ID	bmjopen-2022-062254
Article Type:	Original research
Date Submitted by the Author:	03-Mar-2022

Complete List of Authors:	Tak, Mehroosh; The Royal Veterinary College, Veterinary Epidemiology, Economics and Public Health<br>Law, Cherry; London School of Hygiene & Tropical Medicine, Department of Public Health<br>Green, Rosemary; London School of Hygiene and Tropical Medicine Faculty of Epidemiology and Population Health, Population Health<br>Shankar, Bhavani; University of Sheffield, Department of Geography<br>Cornelsen, Laura; London School of Hygiene & Tropical Medicine, Department of Public Health

Keywords:	NUTRITION & DIETETICS, PUBLIC HEALTH, Health economics < HEALTH SERVICES ADMINISTRATION & MANAGEMENT

SCHOLARONE™
Manuscripts

I, the Submitting Author has the right to grant and does grant on behalf of all authors of the Work (as defined in the below author licence), an exclusive licence and/or a non-exclusive licence for contributions from authors who are: i) UK Crown employees; ii) where BMJ has agreed a CC-BY licence shall apply, and/or iii) in accordance with the terms applicable for US Federal Government officers or employees acting as part of their official duties; on a worldwide, perpetual, irrevocable, royalty-free basis to BMJ Publishing Group Ltd ("BMJ") its licensees and where the relevant Journal is co-owned by BMJ to the co-owners of the Journal, to publish the Work in this journal and any other BMJ products and to exploit all rights, as set out in our licence.

The Submitting Author accepts and understands that any supply made under these terms is made by BMJ to the Submitting Author unless you are acting as an employee on behalf of your employer or a postgraduate student of an affiliated institution which is paying any applicable article publishing charge ("APC") for Open Access articles. Where the Submitting Author wishes to make the Work available on an Open Access basis (and intends to pay the relevant APC), the terms of reuse of such Open Access shall be governed by a Creative Commons licence – details of these licences and which Creative Commons licence will apply to this Work are set out in our licence referred to above.

Other than as permitted in any relevant BMJ Author's Self Archiving Policies, I confirm this Work has not been accepted for publication elsewhere, is not being considered for publication elsewhere and does not duplicate material already published. I confirm all authors consent to publication of this Work and authorise the granting of this licence.

For peer review only

Longitudinal analysis of processed foods purchase profiles for urban Indian households

Mehroosh Tak[1], Cherry Law[2,3], Rosemary Green[4], Bhavani Shankar[5], Laura Cornelsen[2]

[1] Veterinary Epidemiology, Economics and Public Health, The Royal Veterinary College (RVC), Hatfield, UK

[2] Department of Public Health, Environments and Society, London School of Hygiene and Tropical Medicine, London, UK

[3] School of Agriculture, Policy and Development, University of Reading, UK

[4] Department of Population Health, London School of Hygiene and Tropical Medicine, London, UK

[5] Institute for Sustainable Food, University of Sheffield

Corresponding author

Dr Laura Cornelsen, Department of Public Health, Environment and Society, London School of Hygiene & Tropical Medicine, 15-17 Tavistock Place, London, WC1H 9SH, United Kingdom, Laura.Cornelsen@lshtm.ac.uk

Word count: 4690

For peer review only

ABSTRACT

Objective: Sales of ultra-processed foods and beverages (UPF) are rising in low and middle-income countries. Such foods are often linked with weight gain, obesity, type-2 diabetes and hypertension - diseases that are on the rise in India. This paper analysed patterns in purchases of processed and ultra-processed food by urban Indian households.

Setting: Panel data from Kantar – Worldpanel Division, India for 2013 and 2016

Participants: 58,878 urban Indian households

Methods: We used K-mean partition clustering and multivariate regression to analyse patterns in processed food and UPF purchase for urban India.

Results: Three-quarters of urban Indian households purchased over ten processed food groups. Mean per person annual processed food purchase was 150kg. UPF purchase was low at 6.4kg in 2016 but had grown by 6% since 2013. Cluster analysis identified three patterns of consumption, characterised by low (54% of the households in 2016), medium (36%) and high (10%) processed food purchase quantities. High cluster households purchased over three times as much processed foods and UPF as the low cluster households. Notably, salt purchases were persistently high across clusters in both years (>3.3kg), while sweet snack and ready-to-eat food purchases grew consistently in all clusters between 2013 and 2016. A positive and significant association was found between household purchases of UPF and their socioeconomic status as well as ownership of durables, such as refrigerator, colour television and washing machine (all $p<0.001$). Spatial characteristics including size of town ($p<0.05$) in which the household is located were also positively associated with the purchase of UPF.

Conclusion: Results suggest the need for tailored regional and city level interventions to curb the low but growing purchase of UPF. New data on obesity and rise of non-communicable diseases, the results are concerning given the links between lifestyle changes and the speed of urbanisation in Indian cities.

For peer review only

For peer review only - http://bmjopen.bmj.com/site/about/guidelines.xhtml

STRENGTHS AND LIMITATIONS

- Use of a large, objective longitudinal household panel survey of processed food and ultra-processed food (UPF) purchases for 2013 and 2016.
- Representative analysis of all urban India rather than specific cities or regions.
- Multivariate and cluster analysis of the patterns and associations between UPF and socio-economic status and spatial variables.
- The dataset does not include unprocessed food purchases, which would allow for a comparative analysis of dietary transitions towards UPF.
- The survey data collected are for purchases and not consumption of foods.

For peer review only

For peer review only - http://bmjopen.bmj.com/site/about/guidelines.xhtml

1. INTRODUCTION

As India battles the persistent double burden of malnutrition, including rising overweight or obesity rates, the prevalence of non-communicable diseases (NCDs) is posing a significant public health challenge[1]. Recent data from the National Family and Health Survey (NFHS) for 2019-20 reveals that since 2015-16 prevalence of obesity among children under-5 years old increased in 20 out of 22 states[2]. Overweight and obesity have also risen amongst adult population to 21% of women and 19% of men in 2015-16 relative to 13% and 9.3% in 2005-06 respectively[3]. NCDs have long been linked to changing dietary patterns and greater consumption of ultra-processed foods, in particular sweet and salty highly processed snacks and beverages[4, 5]. These changes to diets reflect economic growth and rising disposable incomes for urbanising Indian households[6]. In particular, a global shift towards higher volumes of ultra-processed food and beverages purchases has been documented[7].

While sales of ultra-processed food and beverages is stagnating in high-income countries, it is rapidly rising in middle-income countries[8]. Ultra-processed food are linked with weight gain, obesity, type-2 diabetes and hypertension [9-11] - diseases that are on the rise in India. A systematic review of studies on Indian dietary patterns found an association between high intake of sweets and snacks and higher diabetes risk[12]. Thus, analyses of processed food consumption patterns can be critical to identify the entry points for interventions to prevent diet related diseases and for more targeted public health policy. However, detailed analyses on consumption patterns of processed food and beverages and its socio-economic determinants in India remain a significant gap in the literature.

Thus far, dietary transition analysis in India has primarily relied on the National Survey Statistics Organisation's (NSSO) Household Consumption and Expenditure Survey (HCES) that is known to not capture Indian processed food consumption [13]. Additionally, the last available HCES for India is now almost a decade old (2011-12). Using the HCES for 2011-12, a study found that processed food accounted for almost 10% of the average calorie intake in India [14]. This percentage could be as high as 30% for the richest households in urban India[14]. Since then expenditure on packaged and processed food has almost doubled- per capita sales rose between 2010 and 2020 from USD26.3 to USD59.8 respectively (at constant 2020 prices)[15]. While per capita purchase quantity for processed food in India remains low in comparison to other middle and high income countries, there is considerable variation in dietary patterns across states[13]. Law et al (2019) analysed aggregate trends in purchase of processed food and found rising purchases of sweet and salty snacks in particular. However, this paper did not unpack household level determinants of processed food purchases. Other existing studies rely on small regional data covering limited number of processed food. For example, dietary patterns in Mumbai and Trivandrum showed high intakes of fried snacks and sweets [16]. Another study of dietary patterns among factory workers in tier-2 cities of India, Lucknow, Nagpur, Hyderabad and Bangalore, found two of the three distinct dietary patterns associated with high intake of snacks [17]. A recent study found high incidence of snack food consumption, including bakery products, savoury and sweet snacks among all age groups, gender, socio-economic levels in the 9th largest Indian city of Pune [18].

The aim of this paper is to analyse the patterns of processed, including ultra-processed, food purchases in urban India in greater detail at household level. To do this we use a panel dataset from "Kantar – Worldpanel Division, India" on records of take-home purchases of processed food and beverages in 2013 and 2016[19] from over 60,000 households on 43,237 distinct products. To understand patterns of processed food and beverage purchases we used K-partition cluster analysis and to identify socio-economic determinants of the purchases of ultra-processed food and beverages (UPF) we conducted multivariate regression analysis. As Indian dietary patterns, are influenced by regional, socio-economic and cultural preferences [13, 20] food group purchase analysis was conducted at regional level.

For peer review only - http://bmjopen.bmj.com/site/about/guidelines.xhtml

2. METHODS

2.1. Data

We used data from purchase records of an on-going demographically representative household expenditure panel, collected by the market insight company, "Kantar – Worldpanel Division, India" [19]. Indian households are invited to participate based on their socio-economic status, age of the person responsible for food purchase as well as the sector (urban/rural) and state of domicile. The sampling is based on the 2011 Census. This panel is frequently reviewed by Kantar to assess the need for inviting new households and to ensure its demographic representativeness. Within each participating household, the primary shoppers are asked to record all purchases of processed food taken home daily and to retain all the packaging and wrappers in pre-provided containers. These diaries collect information regarding volume of purchases but not on monetary expenditure or prices. Kantar conducts regular checks over the accuracy of the purchase records by the interviewers, who compare the information in the paper diaries against packaging and wrappers retained by the households. Purchases made for consumption outside of home are not included.

Demographic and socio-economic information for the panel of households is provided with the data for 2013 and 2016. We used purchase records from these two years and aggregate them to annual level to examine temporal changes in processed food purchase across regions. Socio-economic descriptors available included information on household size and composition, socio-economic status (SES), durables owned by households (electricity in the house, ceiling fan, colour television, two wheeler, gas stove, refrigerator, washing machine, laptop or personal computer, four wheeler or air conditioner) and household residence by town size and state. Information of household composition was provided in binary variables indicating if the household includes children who are infant, under 1 year of age, between 2-4 years, 5-9 years, 10-14 years and 15-17 years of age. The SES variable was categorised as upper class (with literacy of at least four years and ownership of at least six durables), upper middle class (literacy of at least four years and ownership of five durables), middle class (literacy of at least four years and ownership of three durables) and lower class (illiterate with up to one durable). Towns were categorised by population size, starting from less than 100,000 people, between 100,000 and 500,000, 0.5-1 million, 1-4million and over 4 million people. Zones were described as East, South, West and North[1].

We created a balanced panel of urban households to allow analysis on temporal change in processed food purchases. The panel retention was high. In 2013 data 64,941 households in urban areas reported purchases, of which 60,274 (93%) were also present in the panel in 2016. Thus, a small percentage of households discontinued participation but the attrition did not show any systematic patterns. We further excluded a small number of households (2%) due to missing information on household size. Our final dataset thus contained annual purchases from a balanced panel of 58,878 urban households.

2.2. Food groups

The 43,237 distinct food items were grouped into 15 processed food (PF) and UPF groups. PF included staples, milk, oils salt, processed wheat, tea & coffee, spices, butters & cheese and salt, while UPF included salty snacks, drinks, ready to eat foods, sweet snacks, milk drinks, frozen foods and breakfast cereals (see supplementary Table 1). UPF were defined as foods that are highly processed and contain

[1] Zonal classification – 1) North - Delhi, Punjab, Haryana and Uttar Pradesh; 2) East - West Bengal, Bihar, Jharkhand, Guwahati (Assam) and Orissa; 3) West - Rajasthan, Maharashtra, Gujarat, Madhya Pradesh, Chhattisgarh; 4) South - Tamil Nadu, Karnataka, Kerala, Andhra Pradesh including Telangana

For peer review only - http://bmjopen.bmj.com/site/about/guidelines.xhtml

in addition to added salt and sugar, additive such as flavours, colouring and emulsifiers which are normally used in industrial processes only[21].

With the exception of milk and drinks for which unit of measure is millilitres (ml), all other food groups were measured in grams (gm). To aggregate the volume of purchases across food groups, we converted the volume for milk and drinks from ml to gm using the conversion rate of 1ml = 1.03 gm. For each year, we also created a food group diversity score (DS) which is the count of number of food groups purchased in that year, ranging from 1 to 15.

2.3. Empirical strategy

2.3.1.K-partition cluster analysis

As a first step, we plotted the distribution of DS across households and describe the average annual purchases of processed food across SES groups. We then compared prevalence and quantity of annual purchases across the regions with a Chi^2 test. Second, we used K-mean partition cluster analysis to group the sampled households into clusters based on similarity of their processed food purchases, allowing identification of distinct and predominant patterns in the data. Clustering was done for both 2013 and 2016 separately to analyse temporality of purchase patterns. K-mean partition uses Euclidean distances between observations to empirically estimate clusters within the dataset[22]. Partition clustering is an iterative process that minimizes within-cluster variability while maximizing between-cluster variability at the same time. The technique assigns observations into a pre-defined number of non-overlapping clusters. Each observation is assigned to the cluster with the closest mean. New cluster means are then calculated after each observation is assigned. The process continues iteratively until no observations change clusters[23].

To run the cluster analysis, we calculated the quantity of foods purchased per household member in each food group by dividing household purchase quantity with household size. Clustering was then conducted for 3 to 8 partitions for each year separately. Once the clusters were constructed, boxplots with confidence intervals were created for each food group by clusters to analyse purchase patterns and determine the best fitting number of clusters. Calinski and Harabasz pseudo-F index was used to identify the appropriate number of clusters which is considered as one of the best rules to apply for this purpose [24]. It was estimated through a function of ([B](g-1))/([W](N-g)), where B is the between-cluster sum of squares and cross-products matrix, W is the within-cluster sum of squares and cross-products matrix, g is the number of cluster groups and N stands for number of observations[25]. The larger the value of pseudo-F the more clearly defined the cluster structures, and vice versa.

2.3.2.Regression analysis

In the final step, pooled ordinary least square (OLS) regression was used to understand the socio-economic determinants of UPF purchases. We used the logarithm of per household member purchase quantity of UPF as the outcome variable and the following explanatory variables: SES, state and town size of residence dummies, household size, binary variables describing household composition of children across ages (under 1 year, 2-4 years, 5-9 years, 10-14 years and 15-17 years) and binary variables describing durable assets owned by the household, including colour TV, refrigerator, washing machine, laptop/ personal computer, four wheeler, air conditioner. We include time fixed effects in the pooled OLS model to control for macroeconomic changes over the data period. All estimations used robust standard errors (SEs) with clustering at state and town population level. This choice was informed by descriptive and cluster analysis where these spatial descriptors showed relevance in differentiating dietary preferences.

For peer review only - http://bmjopen.bmj.com/site/about/guidelines.xhtml

As the SES variable was constructed using education level and ownership of a certain number of durables, we also checked for multicollinearity with binary variables indicating ownership of durables using Variance Inflation Factor (VIF) test. VIF for SES and durables were less than 10 suggesting no issues with multicollinearity. Additionally, we ran regression models together and separately with the SES and durable variables to check if coefficients varied. Our results were robust to these alternative specifications.

2.3.3.Patient and Public Involvement

There are no patients or public participation in this study.

1. RESULTS

1.1. Diversity of processed food purchases

In 2016, three quarters of the households (76%) purchased ten food groups or more out of the 15 used in the study, implying relatively high variety of processed food likely to be consumed by urban Indian households (Supplementary figure 1). Less than 1% of the households purchased all 15 food groups and less than 6% of the households bought seven food groups or less. DS remained constant for 29% of the households, increased for 33% and declined for 37% of the households between 2013 and 2016. Most households purchased less than 10 kgs of UPF per household member. Only 733 households out of 58,878 (1.3%) did not purchase any UPF in 2016 (Supplementary figure 2). The average purchase quantity of UPF in 2016 was 6.4kg.

Figure 1 presents the kernel density curve for annual per household member UPF purchased in kilogram (kg) by SES. It shows that upper and lower class households have more probability weight at low UPF consumption levels compared to middle and upper-middle class households. That is, middle and upper middle class households were more likely to purchase higher quantities of UPF than lower and higher class households. The same kernel density curve for all PF found that households with higher SES were likely to purchase higher quantities of PF (supplementary figure 3).

Figure 1 here

Regional variation in processed food purchaseError! Reference source not found. presents the annual purchase quantities per household member by food group and zones in 2016 and the colours indicate direction of changes between 2013 and 2016. There is considerable variation in purchase patterns across zones. Overall, the purchases were highest in the North zone (218kg annually per household member), followed by West (153kg), East (127kg) and South (108kg). While UPF purchases made up only small share of these, there was an increase in the purchase of UPF overall (by 0.36kg or ~6% p<0.001) and in East (by 1.2kg or ~21% p<0,001) and West (by 0.39kg or ~9% p<0.001) zones.

Table 1: Annual Purchase Quantity (gm) of Individual Food Groups per Household Member by Zone and Change from 2013 to 2016

2016	Per Capita Consumption by Food Group				
Zone	North	East	West	South	Total
Processes Foods					
Staples	65,054 (-364)	39,931 (700)	43,936 (-129)	10,597 (496)***	38,663 (158)
Milk	122,516 (-2,780)**	59,872 (4,923)***	83,980 (2,226)***	74,236 (-2,637)***	84,689 (394)

For peer review only - http://bmjopen.bmj.com/site/about/guidelines.xhtml

Oils	12,848 (-232)*	12,234 (1,277)***	14,600 (154)	10,818 (328)***	12,723 (348)**
Salt	3,243 (-54)*	4,215 (178)***	3,253 (-138)***	4,158 (-114)***	3,691 (-49)***
Processed Wheat	3,058 (-390)***	1,970 (-64)	867 (-133)***	824 (-431)***	1,550 (-254)***
Tea/Coffee	1,052 (-86)***	913 (41)***	1,082 (9)	1,026 (26)**	1,026 (0)
Spices	1,007 (15)	741 (99)***	460 (40)***	593 (104)***	671 (65)***
Butters	241 (0)	103 (-18)***	106 (16)***	79 (-22)***	127 (-5)*
**Total PF Purchases**	**209,020 (-3889)****	**119,979 (7,136)****	**148,284 (2,045)***	**102,330 (-2,251)****	**143,139 (655)**
**Ultra-Processed Foods**					
Salty Snack	1,161 (-10)	833 (184)***	1,296 (84)***	623 (-94)***	991 (36)***
Drinks	4,022 (-116)	1,334 (230)***	856 (-43)	1,600 (-311)***	1,833 (-75)**
Ready to Eat Foods	455 (56)***	425 (84)***	334 (42)***	1,526 (315)***	700 (128)***
Sweet Snacks	2,588 (116)***	3,099 (704)***	2,184 (297)***	1,713 (144)***	2,331 (301)***
Milk Drinks	105 (-28)***	845 (-71)***	91 (-18)***	431 (-87)***	339 (-50)***
Breakfast Cereals	311 (63)***	125 (33)***	101 (24)***	199 (-19)**	177 (22)***
Frozen Foods	33 (-6)**	12 (1)	15 (6)***	6 (-5)***	16 (-1)
**Total UPF purchased**	**8,675 (75)**	**6,674 (1,166)*****	**4,876 (390)*****	**6,098 (-58)**	**6,387 (361)*****
**Total PF + UPF Purchase**	**217,695 (-3,814)****	**126,653 (8,302)*****	**153,160 (2,435)****	**108,428 (-2,309)****	**149,526 (1,016)**
**Diversity Score**	**12 *****	**11**	**10**	**11 *****	**11 *****

Figures in parenthesis show average changes between 2013 and 2016 in grams. *** p-value <0.001, ** p-value <0.01, * p-value <0.05, Cell colour: Green – increase in value, red – decline in value, grey – no change. Beverages were converted from litres to kilograms by multiplying with 1.03.

For peer review only - http://bmjopen.bmj.com/site/about/guidelines.xhtml

Across the food groups, most notably, the per household member purchase of ready to eat foods and sweet snacks has increased across urban India by 128gm (~22%, $p<0.001$) and 301gm (~15%, $p<0.001$) respectively. Purchase of breakfast foods including sugary cereals were very low (0.18kg on average) but also increased considerably (~14%, $p<0.001$). While salt purchase declined marginally from 2013 to 2016, it remains twice as high (3.69 kg per year or 10.1gm per day) compared to recommended 5gm per day [26]. Similarly, oil purchase was high with more than 12.3L purchased per household member per year. Purchase of drinks and milk drinks showed an overall decline by 4% and 13% respectively, although an increase in drinks was seen in the East zone (by 0.223L ~17%).

Quantity of purchases per household member declined most in the North zone, driven by reduction in non-UPF purchases. Regardless, the purchase volume in most foods groups remained highest in the North zone compared to other zones. For example, drinks purchases average to 4.02kg in the North zone, which is more than double of the urban average of 1.83kg. Exceptions were ready-to-eat food purchases which were much higher in the South (1.53kg) followed by North (0.46kg) zone. Other exceptions include milk drinks and sweet snacks that are purchased in greater quantity in the East zone (0.82L and 3.1kg respectively). Per household member purchase of frozen foods was the lowest of all food groups (0.016kg on average).

Finally, North zone also had the highest average DS in 2016 (12/15) which had increased since 2013 ($p<0.001$). This was followed by South and East zone (11/15). West zone had the lowest DS on average (10/15). South zone was the only zone where the decline in average DS was statistically significant ($p<0.001$).

1.2. Purchase Patterns: Cluster Analysis

We ran cluster analysis by year with number of clusters from 3 to 8. The Calinski and Harabasz pseudo-F index (Supplementary table 2 presents index values for each cluster by year) suggested that three clusters were the optimal partitioning for both years. This was further confirmed by visual inspection of cluster purchase patterns in box plots[2]. Reviewing the purchasing patterns in the three partition model, we found that households fell into distinct clusters that were best characterised by purchase quantities rather than purchases of distinct food categories: low purchase, medium purchase and high purchase of processed foods. This clustering patterns was consistent in 2013 and 2016 data.

Means for key variables of interest by clusters by year are presented in **Error! Reference source not found.**. From 2013 to 2016, the share of households in the low cluster declined from 64% to 54% while the proportion of households in medium and high clusters increased from 32% to 36% and from 4% to 10% respectively. Across the years, the quantities purchased were always smallest for low cluster, followed by medium and high cluster, suggesting that clustering patterns did not change over the four-year period. Between 2013 and 2016 overall PF purchase volumes declined in all three clusters, while UPF purchases increased for low ($p<0.01$) and high purchase cluster. Purchases of PF and UPF were more than three times greater in high cluster compared to low cluster. Sweet snacks and ready-to-eat foods were two categories that had consistent increase in all three clusters while milk and milk drinks showed a consistent decrease. Although, low purchase cluster bought less of UPF, the share of UPF in

[2] Due to large number of graphs, these are not presented in the paper but are available upon request.

For peer review only - http://bmjopen.bmj.com/site/about/guidelines.xhtml

their average share of food basket was higher (5.2% in 2016) than medium (3.8%) and high (3.9%) purchase clusters[3]. The difference between cluster means was statistically significant (p<0.001).

The high purchase cluster households were likely to have smaller household size than low and medium purchase clusters (Table 2). In line with this, households in low purchase cluster were more likely to have children in every age category compared to households in middle and high purchase clusters. In term of geographical distribution, households in high purchase cluster were more likely to be from North zone than other zones (58% in 2013 and 49% in 2016). The medium purchase cluster included relatively similar share of households from North and West (38% and 32% respectively in 2013) which remained relatively consistent in 2016. Households from the biggest towns (> 1 million in population) were more likely to be in the high purchase cluster, which may be due to a larger availability of processed food in bigger towns and cities.

Table 2: Processed food purchase patterns for urban India for 2013 and 2016

	2013			2016		
**Purchase Clusters**	**Low**	**Medium**	**High**	**Low**	**Medium**	**High**
Number (%) of HHs	37,331 (63.4%)	19,059 (32.4%)	2,488 (4.2%)	31,883 (54.1%)	21,422 (36.4%)	5,573 (9.5%)
Dietary diversity	10.7	11.1	11.1	10.6	11.0	11.2
Household size	5.1	4.1	2.7	5.0	4.2	2.9
**Average Annual Per Capita Purchase of Food Groups in gm**						
Milk	47,345	129,956	288,926	41,698	111,331	228,233
Staples	28,733	50,734	91,439	24,848	49,069	77,697
Oils	10,913	14,038	21,577	10,725	14,058	19,019
Salt	3,518	3,885	5,953	3,332	3,824	5,235
Processed Wheat	1,309	2,506	3,867	1,060	1,878	3,094
Tea/Coffee	843	1,243	2,115	811	1,168	1,709
Spices	522	687	1,266	572	714	1,069
Butters & Cheese	64	226	426	54	165	402
**Total PF Purchase**	**93,246**	**203,276**	**415,569**	**83,100**	**182,206**	**336,458**
Drinks	918	3,248	6,488	825	2,287	5,849
Sweet Snacks	1,807	2,306	3,266	2,061	2,396	3,623
Salty Snacks	713	1,325	1,752	683	1,208	1,925
Ready to Eat Foods	484	698	930	595	704	1,285
Milk Drinks	361	422	556	320	328	495
Breakfast Cereals	86	246	494	85	228	513
Frozen Food	5	33	69	5	21	55
**Total UPF Purchase**	**4,375**	**8,278**	**13,554**	**4,573**	**7,172**	**13,745**
**% of UPF in Total Purchase**	**4.48%**	**3.91%**	**3.16%**	**5.22%**	**3.79%**	**3.92%**
**Total PF + UPF Purchase**	**97,621**	**211,554**	**429,123**	**87,673**	**189,378**	**350,202**
**Percentage of Households with a Child in Age Group**						
Infant	3%	2%	0%	6%	4%	2%
<1 year	5%	3%	1%	3%	2%	1%
2-4 years	15%	10%	4%	14%	10%	4%
5-9 years	26%	18%	7%	23%	16%	8%
10-14 years	34%	25%	11%	29%	21%	10%

[3] We conducted ANOVA (p-value<0.001) and multivariate test of means (p-value<0.001) to test statistical significance in the difference in UPF purchases by clusters for each year. Null hypothesis of equal means was rejected confirming the difference was significant between clusters.

For peer review only - http://bmjopen.bmj.com/site/about/guidelines.xhtml

15-17 years	26%	19%	8%	23%	18%	9%
**Zone**						
North	11%	38%	58%	9%	33%	49%
East	26%	12%	7%	26%	14%	12%
West	31%	32%	21%	30%	34%	26%
South	32%	18%	14%	34%	20%	13%
**Town size**						
40 Lakhs +	26%	25%	18%	27%	23%	23%
10-40 Lakhs	31%	44%	53%	30%	43%	47%
05-10 Lakhs	15%	11%	7%	15%	11%	9%
01-05 Lakhs	14%	11%	14%	15%	11%	11%
<= 01 Lakhs	14%	10%	8%	14%	10%	10%
**Socio-Economic Status (SES)**						
Lower Class	13%	4%	5%	14%	5%	4%
Middle Class	29%	14%	13%	30%	18%	10%
Upper Middle Class	31%	26%	26%	31%	29%	22%
Upper Class	27%	56%	56%	25%	49%	64%
**Durables/Assets**						
Two Wheeler	53%	70%	66%	58%	70%	69%
Refrigerator	53%	80%	80%	58%	78%	84%
Washing Machine	21%	50%	55%	28%	53%	66%
Four Wheeler	19%	38%	36%	23%	35%	40%
Laptop/Personal Computer	10%	24%	29%	16%	27%	38%
Air Conditioner	5%	16%	24%	6%	17%	34%

As expected, the medium and high purchase clusters contained a greater proportion of households from upper middle and upper class. 56% of households purchasing high quantities of processed food were from upper class in 2013 which increased to 64% in 2016. Furthermore, households purchasing medium and high level of all PF (including UPF) were more likely to own durables such as refrigerators, washing machine, four wheeler, laptop and air conditioner.

Figure 2 presents the share of individual PF (Panel A) and UPF (Panel B) food groups as percentage of total PF and UPF purchases respectively. Panel A shows that the low cluster had a greater share of the foods consumed on a daily basis, such as staples, oils, tea/coffee and spices. For this cluster almost half of the UPF purchases (45%) were sweet snacks (Panel B). This cluster also had the highest share of ready-to-eat foods (13% of UPF) and milk drinks (7% of UPF). In comparison, the high cluster purchased a larger proportion of milk (68% of PF). They also had a higher share of drinks (43% of UPF), breakfast cereals (4% of UPF) and frozen foods (0.4% of UPF). Medium purchase cluster stood out for slightly greater share of salty snacks purchases in comparison to low and high purchase clusters.

Figure 2 here

Finally, we estimated multinomial logit models for groups identified by cluster analysis. The results are presented in supplementary table 3. These results confirmed that higher SES, having older children and durables to be positively and significantly associated with medium and high clusters purchasing higher quantities of all PF than the low cluster.

For peer review only - http://bmjopen.bmj.com/site/about/guidelines.xhtml

1.3. Determinants of Ultra- Processed Food Purchase: Regression analysis

Error! Reference source not found. presents the pooled OLS model for UPF quantity per household member purchased in 2013 and 2016. We found that SES, large town sizes, children under the age fourteen, and durable assets were positively and significantly related to UPF purchase. Of particular importance is the SES. An upper class household purchased 34% ($p<0.001$) ($Y= e^{0.29} = 1.3364$ or 34%) more UPF than a lower class household. Both middle and upper-middle class households purchased more UPF than lower class household by 22% ($p<0.001$) and 14% ($p<0.001$), respectively. Households from towns with more than 4m inhabitants purchased 50% ($p<0.01$) more UPF than households from the smallest towns (population of less than 100,000). In comparison, households in the towns with 1-4m and 0.51m inhabitants purchased 39% ($p<0.01$) and 35% ($p<0.05$) more UPF to households from the smallest towns, respectively. An additional household member was associated with a 13% ($p<0.001$) less UPF purchased per member. Having children under the age of 14 years had a positive (6-9%) and significant association ($p<0.05$) with UPF purchase.

Table 3: Multivariate analysis of UPF purchase quantity (g) per household member (UPF qphm)

**Outcome variable: log(UPF qphm) // Independent Variables**	**Coefficient**	**Standard Error**	**p-value**	**95% CI**	
Base - SES - Lower Class					
SES- Middle Class	0.127	0.032	<0.001	0.062	0.192
SES- Upper Middle Class	0.196	0.043	<0.001	0.109	0.282
SES- Upper Class	0.290	0.049	<0.001	0.193	0.387
Base - Town population <100k					
Town Population - 500k>=X>100k	0.154	0.133	0.252	-0.112	0.421
Town Population - 1mil>=X>500k	0.300	0.129	0.023	0.042	0.557
Town Population - 4mil>=X>1mil	0.331	0.119	0.007	0.094	0.568
Town Population - >4mil	0.402	0.143	0.007	0.117	0.688
Household Size	-0.143	0.005	<0.001	-0.154	-0.132
Infant	0.067	0.028	0.020	0.011	0.122
Children under 1 year	0.080	0.027	0.004	0.027	0.133
Children 2-4 years	0.086	0.011	<0.001	0.065	0.108
Children 5-9 years	0.088	0.013	<0.001	0.062	0.115
Children 10-14 years	0.058	0.010	<0.001	0.038	0.077
Children 15-17 years	0.008	0.011	0.430	-0.013	0.029
Durable: Colour TV	0.183	0.040	<0.001	0.103	0.264
Durable: Refrigerator	0.090	0.024	<0.001	0.043	0.138
Durable: Washing Machine	0.124	0.031	<0.001	0.062	0.186
Durable: Laptop/PC	0.194	0.025	<0.001	0.145	0.243
Durable: Four Wheeler	0.164	0.037	<0.001	0.089	0.239
Durable: Air Conditioner	0.352	0.045	<0.001	0.261	0.443
Time Effect (base 2013)	0.016	0.027	0.563	-0.039	0.070
Constant	1.422	0.152	<0.001	1.119	1.725
Observations	58,878				
State Effect	Yes				

Notes: Pooled OLS with robust standard errors clustered at state and town population level; *** p<0.001, ** p<0.01, * p<0.05

Ownership of durables had a positive and statistically significant association with quantity of UPF purchased. Households that owned an air conditioner purchased 42% ($p<0.001$) more UPF compared to those that did not. Households who owned a computer (laptop or PC) or colour TV were associated

For peer review only - http://bmjopen.bmj.com/site/about/guidelines.xhtml

with 20-21% (p<0.001) more UPF purchase than those households that did not own them (note that only 22% of households owned laptop/PCs but 98% owned a colour TV in 2016). Ownership of a four wheeler and washing machine increased purchase by 18% (p<0.001) and 13% (p<0.001) respectively, while ownership of a refrigerator only increased purchase by 9%. About a third (37%) of the households did not own a refrigerator in 2016 suggesting that households might be primarily purchasing UPF that do not require cold storage.

2. DISCUSSION

This paper used a unique dataset for urban Indian household purchases to assess patterns and socio-economic determinants of processed, and ultra-processed food purchases in 2013 and 2016. We found that three quarters of urban Indian households purchase a higher variety of processed food (10 out of 15 food groups). However, 60% of them purchased processed food at small quantities that was less than 150kgs per household member annually of which vast majority were commonly consumed products such as milk, oil, atta, rice and salt. The average annual purchase of UPF was low at 6.4kg per household member, but increased by 6% between 2013 and 2016. There is significant regional variation in UPF purchase, as North Indian households purchase on average 8.7kg UPF per household member annually. This was followed by East (6.7kg), South (6.1kg) and West (4.9kg) zones. However increase in UPF purchases were notable in East (21%) and West (9%). Northern households also purchased higher quantity of all processed foods. On average they purchased 217kg of processed food per household member in comparison to the national average of 150kg. Southern households purchased less than half of northern average (108kg). A clear health risk is the high average level of annual salt purchases that was at over 3.7kg per person which is nearly twice as high as WHO guideline of 1.8 kg per year[27]. Both quantity and preferences for food groups varied across urban regions in the country.

Cluster analysis found three distinct purchase clusters amongst urban Indian consumers - low (54% of households in 2016), medium (36% of households) and high (10% of households) purchase clusters in 2016. Over time there was a shift towards medium and high purchase clusters with greater share of households accounted by these two clusters compared to 2013. Medium and high purchase clusters were more likely to have households from higher SES and living in big cities. Households in high purchase cluster bought more of every type of food groups, including three times more of UPF than low cluster. However the low purchase cluster had the greatest volume share of more commonly consumed processed food such as rice, atta, oil, tea, coffee, and spices. High purchase cluster had a higher share of higher value processed food such as milk, butter & cheese, drinks, breakfast cereals and frozen foods. Overall purchases of UPF were relatively low, even in the high purchase cluster (e.g. 5.7L of drinks, 3.6kg of sweet snacks and 1.9kg of salty snacks per year) but it was increasing both in high and low purchase clusters. Sweet snacks and ready-to-eat foods in particular showed greatest increase between 2013 and 2016 with both being the two most prominent UPF purchased by low purchase cluster. Finally, we found that quantity of UPF purchased was positively associated with socioeconomic status, town size, and presence of children younger than fourteen as well as ownership of durables, such as refrigerator and a four wheeler vehicle.

Our results are consistent with the limited literature available in this area. For example, Baker and colleagues [7] who used Euromonitor International Passport database to estimate sales of ultra-processed food and beverages found that in 2019, per capita sales of processed food stood at less than 50 kgs in India, while the compounding annual growth rate for sales between 2009-2019 was 6%. Regional differences for dietary patterns were also observed by other studies of Indian diets [13, 20, 28]. For example, using NSSO food purchase data for 2011-12, Western Indian households were found

For peer review only - http://bmjopen.bmj.com/site/about/guidelines.xhtml

to have lower dietary diversity score than rest of the country [20]. Our analysis also found diversity of processed food purchased to be one group lower for households in Western zone, on average. A recent study based on 24hour recall data for North and South Indian households found salt intake to be 11gms per day or 4kgs annually, similarly to our findings [29]. The study also found that salt intake was mainly from added salt during cooking. Our results confirm that in comparison to Latin American countries, purchase of sugar-sweetened beverages in India is relatively low [30]. The positive association between processed food consumption and socioeconomic position has been also observed in other countries [31-34].

Key limitation of this paper is that the dataset did not include unprocessed food purchases, which would allow a more refined analysis of dietary transitions towards processed and ultra-processed foods. In future should new NSSO data still lack detail on processed food a useful avenue for research could be a matching analysis of NSSO and Kantar data. Previous analysis has shown that the two data sources provide very similar purchase estimates for products present in both (e.g. oil, spices, soft drinks) [13].

Another limitation, also inevitable due to lack of data, is the missing information of out-of-home purchases which include purchases made on-the-go as well as food consumed in restaurants, cafes or work and study places. Additionally, we do not know the exact composition of the household in terms of age and gender and therefore have to assume that food and beverage purchases are shared equally across household members. Finally, Kantar data is based on purchase of food items rather than actual consumption. These limitations however, are not unique to these data. Research using similar granular purchase data in high-income countries also make this assumption[35]. Regardless of the limitations, the analysis helps to improve existing evidence on the nature of processed food in urban Indian diets, which are often linked to increasing obesity and diet-related non-communicable diseases.

Our findings underline important differences and changes in dietary patterns over time in urban Indian population. The results have critical implications for ongoing debates on the role of processed and ultra-processed food in low and middle income countries. Key concerns are low but rising purchase of UPF, persistently high levels of salt purchase and growing trends towards sweet and salty snacks, breakfast cereals and ready-to-eat foods across the country but particularly in Northern India. Significant role of socio-economic status, town size and regional preferences suggest the need for tailored regional and city level interventions to curb the low but growing purchase of UPF. The results are concerning given the links between lifestyle changes and speed of urbanisation [36] especially in tier-2 and 3 cities of the country along with the recently released NFHS survey results for 2019-20 that found a dramatic rise in obesity among children under five in 20 out of 22 Indian states [37].

For peer review only - http://bmjopen.bmj.com/site/about/guidelines.xhtml

3. Data availability statement

Kantar data is proprietary data and thus cannot be made publicly available. Requests regarding data availability should be made to Kantar Worldpanel Division India.

4. Ethics statement

Ethics approval was not required as the study uses secondary anonymised data

5. Competing Interests

None declared.

6. Funding

This study forms part of the Sustainable and Healthy Food Systems (SHEFS) programme supported by the Wellcome Trust's Our Planet, Our Health programme [grant number: 205200/Z/16/Z]. LC is funded by a UK Medical Research Council fellowship (grant number MR/P021999/1).

7. Contributorship Statement

MT, LC, CL designed the study. MT conducted the data analysis. MT, LC, CL, RG and BS interpreted the data and the analysis findings. MT wrote the first draft. CL, LC, BS and RG critically revised and edited the manuscript for intellectual content. All authors have read and approved the final draft.

For peer review only

For peer review only - http://bmjopen.bmj.com/site/about/guidelines.xhtml

References

1. ICMR and PHFI, *Health of the Nation's States—The India State-Level Disease Burden Initiative. New Dehli: Indian Council of Medical Research.* 2017, Institute for Health Metrics and Evaluation, Public Health Foundation of India.
2. Economic Times, *Unhealthy diet, lack of physical activities lead to drastic rise in obesity among adults and kids Men and women with BMI over or equal to 25.0 kg/m2 were counted as obese*, in *Economic Times*. 2020: New Delhi.
3. IIPS and ICF, *National Family Health Survey (NFHS-4), 2015-16*. 2017, International Institute for Population Sciences India. Mumbai.
4. Popkin, B.M., L.S. Adair, and S.W. Ng, *Global nutrition transition and the pandemic of obesity in developing countries.* Nutr Rev, 2012. **70**(1): p. 3-21.
5. Garnett, T., *Plating up solutions.* 2016. **353**(6305): p. 1202-1204.
6. Bren d'Amour, C., et al., *Urbanization, processed foods, and eating out in India.* Global Food Security, 2020. **25**: p. 100361.
7. Baker, P., et al., *Ultra-processed foods and the nutrition transition: Global, regional and national trends, food systems transformations and political economy drivers.* Obesity Reviews, 2020. **21**(12): p. e13126.
8. Moodie, R., et al., *Ultra-Processed Profits: The Political Economy of Countering the Global Spread of Ultra-Processed Foods – A Synthesis Review on the Market and Political Practices of Transnational Food Corporations and Strategic Public Health Responses.* International Journal of Health Policy and Management, 2021.
9. Mendonça, R.D., et al., *Ultra-Processed Food Consumption and the Incidence of Hypertension in a Mediterranean Cohort: The Seguimiento Universidad de Navarra Project.* Am J Hypertens, 2017. **30**(4): p. 358-366.
10. Mendonça, R.D., et al., *Ultraprocessed food consumption and risk of overweight and obesity: the University of Navarra Follow-Up (SUN) cohort study.* Am J Clin Nutr, 2016. **104**(5): p. 1433-1440.
11. Srour, B., et al., *Ultraprocessed Food Consumption and Risk of Type 2 Diabetes Among Participants of the NutriNet-Santé Prospective Cohort.* JAMA Intern Med, 2020. **180**(2): p. 283-291.
12. Green, R., et al., *Dietary patterns in India: a systematic review.* Br J Nutr, 2016. **116**(1): p. 142-8.
13. Law, C., et al., *Purchase trends of processed foods and beverages in urban India.* Glob Food Sec, 2019. **23**: p. 191-204.
14. Sharma, M., et al., *A comparison of the Indian diet with the EAT-Lancet reference diet.* BMC Public Health, 2020. **20**(1): p. 812.
15. Euromonitor, *Passport Global Market Information Database*, E. International, Editor. 2020: London.
16. Daniel, C.R., et al., *A cross-sectional investigation of regional patterns of diet and cardio-metabolic risk in India.* Nutr J, 2011. **10**: p. 12.
17. Satija, A., et al., *Dietary patterns in India and their association with obesity and central obesity.* Public Health Nutr, 2015. **18**(16): p. 3031-41.
18. Roy, D., et al., *Snack Food Consumption across the Pune Transect in India: A Comparison of Dietary Behaviors Based on Consumer Characteristics and Locations.* 2021. **13**(12): p. 4325.
19. Kantar, *Worldpanel Division, India*, Kantar, Editor. 2018.
20. Tak, M., B. Shankar, and S. Kadiyala, *Dietary Transition in India: Temporal and Regional Trends, 1993 to 2012.* Food and Nutrition Bulletin, 2019. **40**(2): p. 254-270.
21. Monteiro, C.A., et al., *Ultra-processed foods: what they are and how to identify them.* Public Health Nutrition, 2019. **22**(5): p. 936-941.
22. Jacobson, H.N. and J.L. Stanton, *Pattern analysis in nutrition.* Clinical nutrition, 1986. **5**: p. 249–253.

For peer review only - http://bmjopen.bmj.com/site/about/guidelines.xhtml

23. Aldenderfer, M.S. and R.K. Blashfield, *Cluster Analysis. Quantitative Applications in the Social Sciences*. 1984, Newbury Park, CA: Sage Publications.
24. Milligan, G.W. and M.C. Cooper, *An examination of procedures for determining the number of clusters in a data set.* Psychometrika, 1985. **50**(2): p. 159-179.
25. Caliński, T. and J. Harabasz, *A dendrite method for cluster analysis.* Communications in Statistics, 1974. **3**(1): p. 1-27.
26. WHO, *Salt reduction*, in *Factsheets*. 2020, WHO.
27. WHO, *Report of the Formal Meeting of Member States to conclude the work on the comprehensive global monitoring framework, including indicators, and a set of voluntary global targets for the prevention and control of communicable diseases*. 2012.
28. Choudhury, S., et al., *What underlies inadequate and unequal fruit and vegetable consumption in India? An exploratory analysis.* Global Food Security, 2020. **24**: p. 100332.
29. Johnson, C., et al., *Sources of Dietary Salt in North and South India Estimated from 24 Hour Dietary Recall.* Nutrients, 2019. **11**(2): p. 318.
30. Popkin, B.M. and C. Hawkes, *Sweetening of the global diet, particularly beverages: patterns, trends, and policy responses.* The lancet. Diabetes & endocrinology, 2016. **4**(2): p. 174-186.
31. Moubarac, J.C., et al., *Consumption of ultra-processed foods and likely impact on human health. Evidence from Canada.* Public Health Nutr, 2013. **16**(12): p. 2240-8.
32. Djupegot, I.L., et al., *The association between time scarcity, sociodemographic correlates and consumption of ultra-processed foods among parents in Norway: a cross-sectional study.* BMC Public Health, 2017. **17**(1): p. 447.
33. Khandpur, N., et al., *Sociodemographic factors associated with the consumption of ultra-processed foods in Colombia.* Revista de saude publica, 2020. **54**: p. 19-19.
34. Simões, B.D.S., et al., *Consumption of ultra-processed foods and socioeconomic position: a cross-sectional analysis of the Brazilian Longitudinal Study of Adult Health (ELSA-Brasil).* Cad Saude Publica, 2018. **34**(3): p. e00019717.
35. Scheelbeek, P.F.D., et al., *Potential impact on prevalence of obesity in the UK of a 20% price increase in high sugar snacks: modelling study.* 2019. **366**: p. l4786.
36. Misra, A., et al., *Nutrition transition in India: secular trends in dietary intake and their relationship to diet-related non-communicable diseases.* J Diabetes, 2011. **3**(4): p. 278-92.
37. Sciences, I.I.f.P., *National Family Health Survey (NFHS-5) 2019–2020 fact sheets: Key Indicators 22 States/UTs from phase I*, M.o.H.a.F.W. International Institute of Population Sciences, Government of India. , Editor. 2020.

For peer review only

For peer review only - http://bmjopen.bmj.com/site/about/guidelines.xhtml

8. Figure legend

Figure 1: Kernel Density Curves for UPF Purchase by Socio-Economic Status in 2016

Figure 2: Share of Food Groups Purchase Quantity by Clusters in 2016

For peer review only

For peer review only - http://bmjopen.bmj.com/site/about/guidelines.xhtml

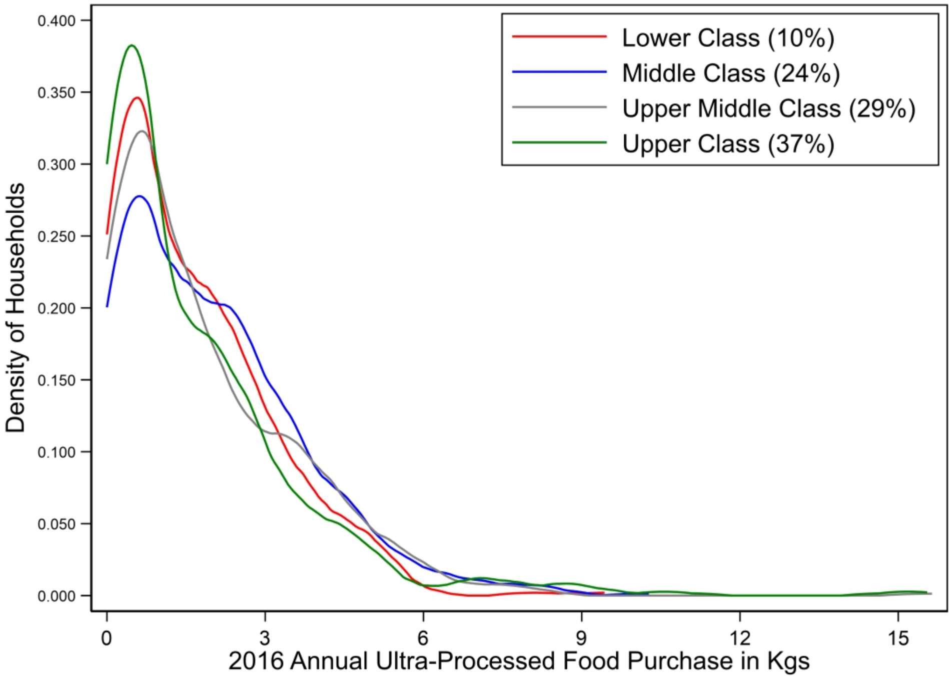

Figure 1: Kernel Density Curves for UPF Purchase by Socio-Economic Status in 2016

2205x1604mm (38 x 38 DPI)

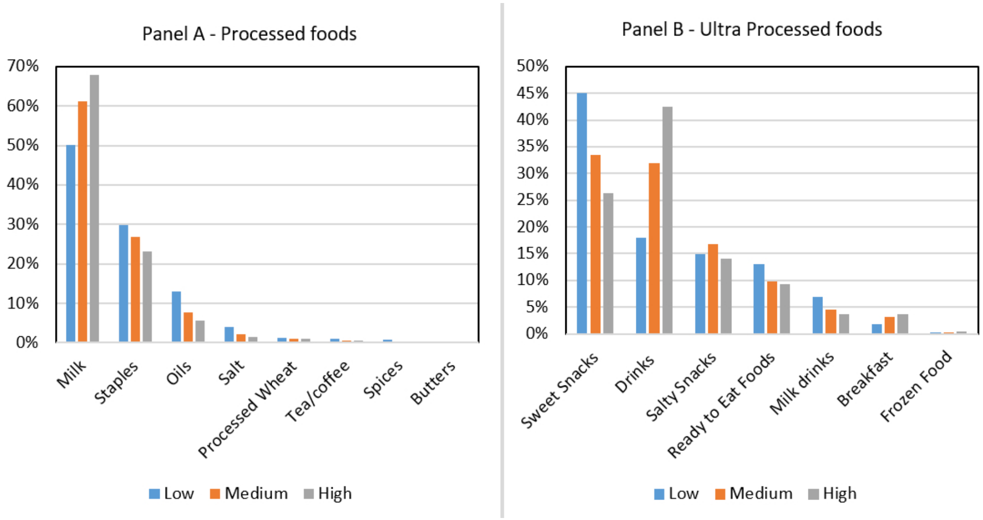

Figure 2: Share of Food Groups Purchase Quantity by Clusters in 2016

734x378mm (38 x 38 DPI)

Supplementary Appendix

{ dzLJLJ f Ŝ Ÿ Ŝ ƴ ű ŀ NJ è ¢ ŀ ō f Ŝ м Y C 2 2 Ř DNJ 2 dzLJ ă

	Food group	Individual food items
1	Staples	Basmati rice [a], atta
2	Milk	Milk
3	Oils	Edible oil, ghee, vanaspati
4	Salt	Salt
5	Processed Wheat	Pasta, bread
6	Tea and Coffee	Tea, coffee
7	Spices	Spices
8	Butters and Cheese	Butter, cheese
9	Salty Snacks (UPF)	Chips, collet, popcorn, potato chips, puffed snacks, traditional snacks
10	Drinks (UPF)	Carbonated drinks, juices, milk based drink, squash
11	Ready to Eat Foods (UPF)	Cooking paste, cook mix, noodle, soup, sauces, pickles, ready meals
12	Sweet Snacks (UPF)	Biscuits, rusk, chocolate, chocolate spread, peanut, butter, jams
13	Milk Drinks (UPF)	Milk food drinks (e.g. malt drinks, chocolate milk), Milk powder (including infant milk powder)
14	Frozen Foods (UPF)	Frozen foods (e.g. fish fingers, samosas)
15	Breakfast cereals (UPF)	Breakfast cereals

Note: [a] Includes data on basmati rice only, which is a higher quality rice and thus does not include the non-basmati variety of rice that is consumed by most Indians

{ dzLJLJ f Ŝ Ÿ Ŝ ƴ ű ŀ NJ è ¢ ŀ ō f Ŝ н Y / ŀ f ƛ ƴ ă ʅ ƛ ŀ ƴ Ř I ŀ NJ ŀ ō ŀ ă Î LJ ă

Clusters	2013	2016
3	37,991.23	41,112.57
4	36,085.22	37,939.35
5	37,141.41	37,687.36
6	35,393.65	36,583.87
7	36,005.66	35,706.64
8	34,889.70	34,865.53

{ dzLJLJ f Ŝ Ÿ Ŝ ƴ ű ŀ NJ è ¢ ŀ ō f Ŝ о Y / f dz ă ű Ŝ NJ Ÿ dz f ű ƛ ƴ 2 Ÿ ƛ ŀ f f 2 Ǝ ƛ

	Cluster	
VARIABLES	2013	2016
Base Cluster Low		
Cluster - Medium		
SES- Middle Class	1.388*** (0.102)	1.548*** (0.097)
SES- Upper Middle Class	2.133*** (0.247)	2.243*** (0.23)
SES- Upper Class	3.526*** (0.531)	3.792*** (0.426)
Town Population - 500k>=X>100k	1.048 (0.161)	0.979 (0.147)
Town Population - 1mil>=X>500k	0.934 (0.159)	1.008 (0.172)
Town Population - 4mil>=X>1mil	1.041 (0.191)	0.973 (0.167)

For peer review only

Town Population - >4mil	0.835 (0.217)	0.756 (0.173)
Household Size	0.542*** (0.017)	0.564*** (0.022)
Infant	1.195* (0.089)	1.089 (0.098)
Children under 1 year	1.001 (0.067)	1.042 (0.099)
Children 2-4 years	1.044 (0.052)	0.996 (0.039)
Children 5-9 years	0.883*** (0.029)	0.898* (0.04)
Children 10-14 years	0.836*** (0.027)	0.837*** (0.023)
Children 15-17 years	0.706*** (0.028)	0.805*** (0.024)
Durable: Colour TV	1.17* (0.086)	1.5*** (0.137)
Durable: Refrigerator	1.534*** (0.074)	1.358*** (0.074)
Durable: Washing Machine	1.45*** (0.1)	1.412*** (0.115)
Durable: Laptop/PC	1.271*** (0.069)	1.171** (0.058)
Durable: Four Wheeler	1.483** (0.179)	1.288** (0.122)
Durable: AC	1.846*** (0.216)	1.816*** (0.201)
Constant	5.826*** (1.307)	7.4*** (1.767)
Cluster - High		
SES- Middle Class	1.546* (0.283)	1.665*** (0.214)
SES- Upper Middle Class	3.278*** (0.691)	3.64*** (0.617)
SES- Upper Class	5.87*** (1.507)	9.556*** (1.95)
Town Population - 500k>=X>100k	1.35 (0.473)	0.808 (0.193)
Town Population - 1mil>=X>500k	0.629 (0.23)	0.718 (0.181)
Town Population - 4mil>=X>1mil	1.143 (0.321)	0.742 (0.201)
Town Population - >4mil	0.892 (0.418)	0.436* (0.181)
Household Size	0.157*** (0.025)	0.174*** (0.018)
Infant	0.923 (0.297)	1.289 (0.229)
Children under 1 year	1.403 (0.373)	0.996 (0.308)
Children 2-4 years	1.2 (0.255)	1.104 (0.168)
Children 5-9 years	0.747** (0.072)	0.981 (0.085)
Children 10-14 years	0.804 (0.103)	0.734** (0.067)
Children 15-17 years	0.486*** (0.054)	0.731*** (0.051)
Durable: Color TV	1.668*** (0.23)	2.714*** (0.476)
Durable: Refrigerator	1.435** (0.17)	1.428** (0.16)
Durable: Washing Machine	1.597** (0.215)	1.681*** (0.194)
Durable: Laptop/PC	1.478** (0.185)	1.335** (0.113)
Durable: Four Wheeler	2.208*** (0.439)	1.916*** (0.26)
Durable: AC	3.199*** (0.844)	3.433*** (0.552)
Constant	15.488*** (7.106)	38.817*** (17.155)
Observations	58,878	58,878
State Effect	Yes	Yes

Notes: Robust standard errors in parentheses; *** p<0.001, ** p<0.01, * p<0.5

For peer review only

{ dzLJLJ ƒ Ŝ Ƴ Ŝ ƴ ű ŀ NJ è C ƛ Ǝ dzNJ Ŝ м Y 5 ƛ ă ű NJ ƛ ō dz ű ƛ ƻ ƴ ƻ Ŧ t NJ ƻ Ǒ Ŝ ă ă Ŝ

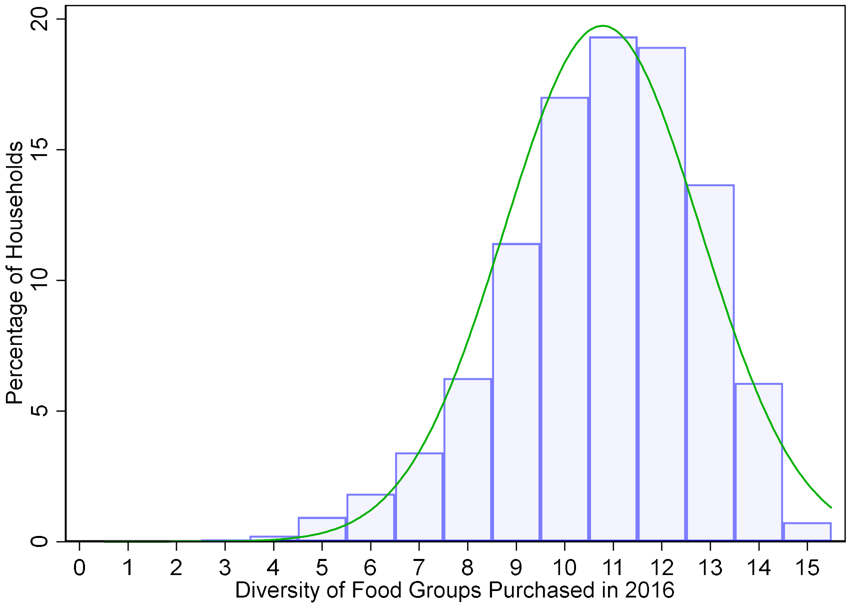

{ dzLJLJ ƒ Ŝ Ƴ Ŝ ƴ ű ŀ NJ è C ƛ Ǝ dzNJ Ŝ н Y 5 ƛ ă ű NJ ƛ ō dz ű ƛ ƻ ƴ ƻ Ŧ ŀ ƴ ƴ dz ŀ ƒ

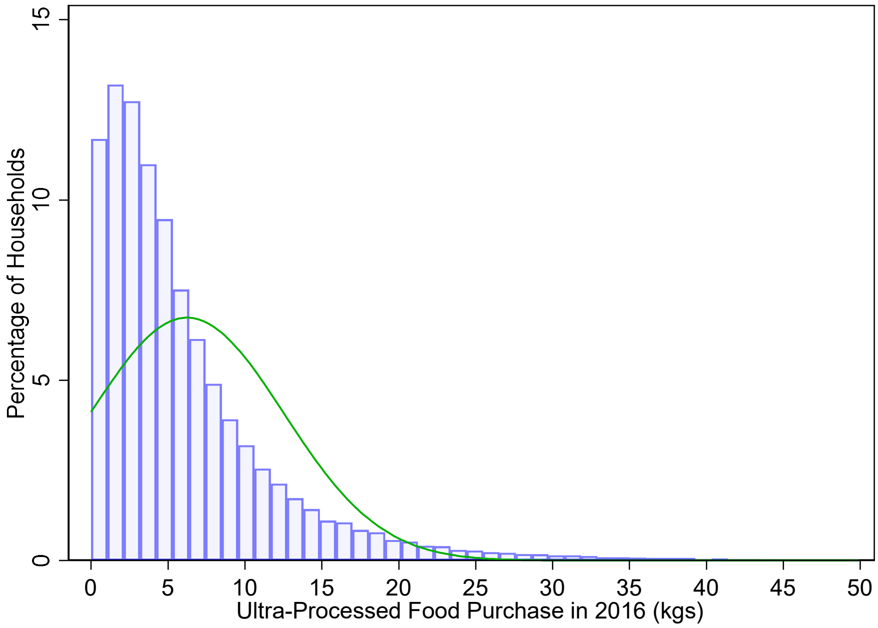

Notes: For readability purposes, the distribution excludes 140 households (0.2%) that purchased more than 50kgs of UPF per household member.

[illegible]

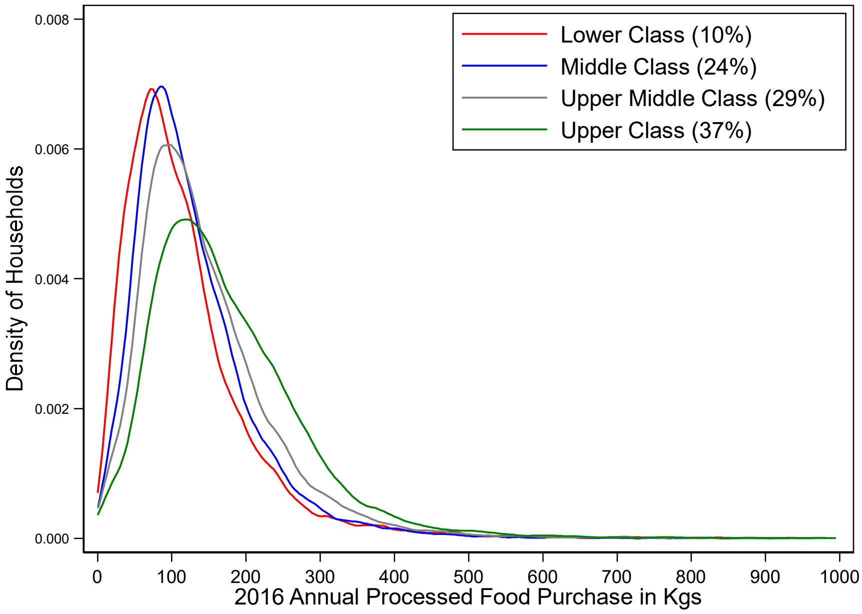

er review only

BMJ Open

Processed foods purchase profiles in urban India in 2013 and 2016: a cluster and multivariate analysis

Journal:	*BMJ Open*
Manuscript ID	bmjopen-2022-062254.R1
Article Type:	Original research
Date Submitted by the Author:	23-Aug-2022

Complete List of Authors:	Tak, Mehroosh; The Royal Veterinary College, Veterinary Epidemiology, Economics and Public Health<br>Law, Cherry; London School of Hygiene & Tropical Medicine, Department of Public Health<br>Green, Rosemary; London School of Hygiene and Tropical Medicine Faculty of Epidemiology and Population Health, Population Health<br>Shankar, Bhavani; University of Sheffield, Department of Geography<br>Cornelsen, Laura; London School of Hygiene & Tropical Medicine, Department of Public Health

**Primary Subject Heading**:	Public health
Secondary Subject Heading:	Health economics
Keywords:	NUTRITION & DIETETICS, PUBLIC HEALTH, Health economics < HEALTH SERVICES ADMINISTRATION & MANAGEMENT

SCHOLARONE™
Manuscripts

BMJ

I, the Submitting Author has the right to grant and does grant on behalf of all authors of the Work (as defined in the below author licence), an exclusive licence and/or a non-exclusive licence for contributions from authors who are: i) UK Crown employees; ii) where BMJ has agreed a CC-BY licence shall apply, and/or iii) in accordance with the terms applicable for US Federal Government officers or employees acting as part of their official duties; on a worldwide, perpetual, irrevocable, royalty-free basis to BMJ Publishing Group Ltd ("BMJ") its licensees and where the relevant Journal is co-owned by BMJ to the co-owners of the Journal, to publish the Work in this journal and any other BMJ products and to exploit all rights, as set out in our licence.

The Submitting Author accepts and understands that any supply made under these terms is made by BMJ to the Submitting Author unless you are acting as an employee on behalf of your employer or a postgraduate student of an affiliated institution which is paying any applicable article publishing charge ("APC") for Open Access articles. Where the Submitting Author wishes to make the Work available on an Open Access basis (and intends to pay the relevant APC), the terms of reuse of such Open Access shall be governed by a Creative Commons licence – details of these licences and which Creative Commons licence will apply to this Work are set out in our licence referred to above.

Other than as permitted in any relevant BMJ Author's Self Archiving Policies, I confirm this Work has not been accepted for publication elsewhere, is not being considered for publication elsewhere and does not duplicate material already published. I confirm all authors consent to publication of this Work and authorise the granting of this licence.

For peer review only

Processed foods purchase profiles in urban India in 2013 and 2016: a cluster and multivariate analysis

Mehroosh Tak[1], Cherry Law[2,3], Rosemary Green[4], Bhavani Shankar[5], Laura Cornelsen[2]

[1] Veterinary Epidemiology, Economics and Public Health, The Royal Veterinary College (RVC), Hatfield, UK

[2] Department of Public Health, Environments and Society, London School of Hygiene and Tropical Medicine, London, UK

[3] School of Agriculture, Policy and Development, University of Reading, UK

[4] Department of Population Health, London School of Hygiene and Tropical Medicine, London, UK

[5] Institute for Sustainable Food, University of Sheffield

Corresponding author

Dr Laura Cornelsen, Department of Public Health, Environment and Society, London School of Hygiene & Tropical Medicine, 15-17 Tavistock Place, London, WC1H 9SH, United Kingdom, Laura.Cornelsen@lshtm.ac.uk

Word count: 4690

For peer review only

ABSTRACT

Objective: Sales of ultra-processed foods and beverages (UPF) are rising in low and middle-income countries. Such foods are often linked with weight gain, obesity, type-2 diabetes and hypertension - diseases that are on the rise in India. This paper analysed patterns in purchases of processed and ultra-processed food by urban Indian households.

Setting: Panel data from Kantar – Worldpanel Division, India for 2013 and 2016

Participants: 58,878 urban Indian households

Methods: We used K-mean partition clustering and multivariate regression to analyse patterns in processed food and UPF purchase for urban India.

Results: Three-quarters of urban Indian households purchased over ten processed food groups. Mean per person annual processed food purchase was 150kg. UPF purchase was low at 6.4kg in 2016 but had grown by 6% since 2013. Cluster analysis identified three patterns of consumption, characterised by low (54% of the households in 2016), medium (36%) and high (10%) processed food purchase quantities. High cluster households purchased over three times as much processed foods and UPF as the low cluster households. Notably, salt purchases were persistently high across clusters in both years (>3.3kg), while sweet snack and ready-to-eat food purchases grew consistently in all clusters between 2013 and 2016. A positive and significant association was found between household purchases of UPF and their socioeconomic status as well as ownership of durables, such as refrigerator, colour television and washing machine (all $p<0.001$). Spatial characteristics including size of town ($p<0.05$) in which the household is located were also positively associated with the purchase of UPF.

Conclusion: Results suggest the need for tailored regional and city level interventions to curb the low but growing purchase of UPF. New data on obesity and rise of non-communicable diseases, the results are concerning given the links between lifestyle changes and the speed of urbanisation in Indian cities.

For peer review only

STRENGTHS AND LIMITATIONS

- Use of a large, objective longitudinal household panel survey of processed food and ultra-processed food (UPF) purchases for 2013 and 2016.
- Representative analysis of all urban India rather than specific cities or regions.
- Multivariate and cluster analysis of the patterns and associations between UPF and socio-economic status and spatial variables.
- The dataset does not include unprocessed food purchases, which would allow for a comparative analysis of dietary transitions towards UPF.
- The survey data collected are for purchases and not consumption of foods.

For peer review only

For peer review only - http://bmjopen.bmj.com/site/about/guidelines.xhtml

1. INTRODUCTION

As India battles the persistent double burden of malnutrition, including rising overweight or obesity rates, the prevalence of non-communicable diseases (NCDs) is posing a significant public health challenge[1]. Recent data from the National Family and Health Survey (NFHS) for 2019-20 reveals that since 2015-16 prevalence of obesity among children under-5 years old increased in 20 out of 22 states[2]. Overweight and obesity have also risen amongst adult population to 21% of women and 19% of men in 2015-16 relative to 13% and 9.3% in 2005-06 respectively[3]. NCDs have long been linked to changing dietary patterns and greater consumption of ultra-processed foods, in particular sweet and salty highly processed snacks and beverages[4, 5]. These changes to diets reflect economic growth and rising disposable incomes for urbanising Indian households[6]. In particular, a global shift towards higher volumes of ultra-processed food and beverages purchases has been documented[7].

While sales of ultra-processed food and beverages is stagnating in high-income countries, it is rapidly rising in middle-income countries[8]. Ultra-processed food are linked with weight gain, obesity, type-2 diabetes and hypertension [9-11] - diseases that are on the rise in India. A systematic review of studies on Indian dietary patterns found an association between high intake of sweets and snacks and higher diabetes risk[12]. Thus, analyses of processed food consumption patterns can be critical to identify the entry points for interventions to prevent diet related diseases and for more targeted public health policy. However, detailed analyses on consumption patterns of processed food and beverages and its socio-economic determinants in India remain a significant gap in the literature.

Thus far, dietary transition analysis in India has primarily relied on the National Survey Statistics Organisation's (NSSO) Household Consumption and Expenditure Survey (HCES) that is known to not capture Indian processed food consumption [13]. Additionally, the last available HCES for India is now almost a decade old (2011-12). Using the HCES for 2011-12, a study found that processed food accounted for almost 10% of the average calorie intake in India [14]. This percentage could be as high as 30% for the richest households in urban India[14]. Since then expenditure on packaged and processed food has almost doubled- per capita sales rose between 2010 and 2020 from USD26.3 to USD59.8 respectively (at constant 2020 prices)[15]. While per capita purchase quantity for processed food in India remains low in comparison to other middle and high income countries, there is considerable variation in dietary patterns across states[13]. Law et al (2019) analysed aggregate trends in purchase of processed food and found rising purchases of sweet and salty snacks in particular. However, this paper did not unpack household level determinants of processed food purchases. Other existing studies rely on small regional data covering limited number of processed food. For example, dietary patterns in Mumbai and Trivandrum showed high intakes of fried snacks and sweets [16]. Another study of dietary patterns among factory workers in tier-2 cities of India, Lucknow, Nagpur, Hyderabad and Bangalore, found two of the three distinct dietary patterns associated with high intake of snacks [17]. A recent study found high incidence of snack food consumption, including bakery products, savoury and sweet snacks among all age groups, gender, socio-economic levels in the 9th largest Indian city of Pune [18].

The aim of this paper is to analyse the patterns of processed, including ultra-processed, food purchases in urban India in greater detail at household level. To do this we use a panel dataset from "Kantar – Worldpanel Division, India" on records of take-home purchases of processed food and beverages in 2013 and 2016[19] from over 60,000 households on 43,237 distinct products. To understand patterns of processed food and beverage purchases we used K-partition cluster analysis and to identify socio-economic determinants of the purchases of ultra-processed food and beverages (UPF) we conducted multivariate regression analysis. As Indian dietary patterns, are influenced by

For peer review only - http://bmjopen.bmj.com/site/about/guidelines.xhtml

regional, socio-economic and cultural preferences [13, 20] food group purchase analysis was conducted at regional level.

2. METHODS

2.1. Data

We used data from purchase records of an on-going demographically representative household expenditure panel, collected by the market insight company, "Kantar – Worldpanel Division, India" [19]. Commercially collected data on food purchases have been increasingly used in academic research given their high frequency and high level of disaggregation. In particular, the household food purchase data collected by Kantar Worldpanel in the UK, Chile, Mexico and South Africa have been recently used to evaluate the effectiveness of food taxes and marketing regulations [21-25]. A recent systematic review concluded that commercially collected data on food purchases are a good indicator of diet at population level and particularly useful for measuring dietary patterns in countries that do not have national dietary surveys carried out regularly, such as India [26].

This panel, which has been operating since 1981, covers 131 towns in 17 urban states in India. There was a major update after the 2011 Indian Census to ensure the panel's representativeness to the urban population with respect to the state of domicile, age of the person responsible for food purchase as well as socio-economic status. Indian households are sampled door-to-door and invited to participate based on these demographic characteristics. The panel is frequently reviewed by Kantar to assess the need for inviting new households and to ensure its representativeness of the 2011 Indian Census. Within each participating household, the primary shoppers are asked to record all purchases of processed food taken home daily and to retain all the packaging and wrappers in pre-provided containers. These diaries collect information regarding volume of purchases but not on monetary expenditure or prices. Kantar conducts regular checks over the accuracy of the purchase records by the interviewers, who compare the information in the paper diaries against packaging and wrappers retained by the households as well as existing products in pantry to avoid double counting.. Purchases made for consumption outside of home are not included in these data.

Demographic and socio-economic information for the panel of households is provided with the data for 2013 and 2016. We used purchase records from these two years and aggregate them to annual level to examine temporal changes in processed food purchase across regions. Socio-economic descriptors available included information on household size and composition, socio-economic status (SES), durables owned by households (electricity in the house, ceiling fan, colour television, two wheeler, gas stove, refrigerator, washing machine, laptop or personal computer, four wheeler or air conditioner) and household residence by town size and state. Information of household composition was provided in binary variables indicating if the household includes children who are infant, under 1 year of age, between 2-4 years, 5-9 years, 10-14 years and 15-17 years of age. The SES variable was categorised as upper class (with literacy of at least four years and ownership of at least six durables), upper middle class (literacy of at least four years and ownership of five durables), middle class (literacy of at least four years and ownership of three durables) and lower class (illiterate with up to one durable). Towns were categorised by population size, starting from less than 100,000 people, between 100,000 and 500,000, 0.5-1 million, 1-4million and over 4 million people. Zones were described as East, South, West and North[1].

---

[1] Zonal classification – 1) North - Delhi, Punjab, Haryana and Uttar Pradesh; 2) East - West Bengal, Bihar, Jharkhand, Guwahati (Assam) and Orissa; 3) West - Rajasthan, Maharashtra, Gujarat, Madhya Pradesh, Chhattisgarh; 4) South - Tamil Nadu, Karnataka, Kerala, Andhra Pradesh including Telangana

For peer review only - http://bmjopen.bmj.com/site/about/guidelines.xhtml

We created a balanced panel of urban households to allow analysis on temporal change in processed food purchases. The panel retention was high. In 2013 data 64,941 households in urban areas reported purchases, of which 60,274 (93%) were also present in the panel in 2016. Thus, a small percentage of households discontinued participation but the attrition did not show any systematic patterns. We further excluded a small number of households (2%) due to missing information on household size. Our final dataset thus contained annual purchases from a balanced panel of 58,878 urban households.

2.2. Food groups

The 43,237 distinct food items were grouped into 15 processed food (PF) and UPF groups. PF included staples, milk, oils salt, processed wheat, tea & coffee, spices, butters & cheese and salt, while UPF included salty snacks, drinks, ready to eat foods, sweet snacks, milk drinks, frozen foods and breakfast cereals (see supplementary Table 1). UPF were defined as foods that are highly processed and contain in addition to added salt and sugar, additive such as flavours, colouring and emulsifiers which are normally used in industrial processes only[27].

With the exception of milk and drinks for which unit of measure is millilitres (ml), all other food groups were measured in grams (gm). To aggregate the volume of purchases across food groups, we converted the volume for milk and drinks from ml to gm using the conversion rate of 1ml = 1.03 gm. For each year, we also created a food group diversity score (DS) which is the count of number of food groups purchased in that year, ranging from 1 to 15.

2.3. Empirical strategy

2.3.1. K-mean partition cluster analysis

As a first step, we plotted the distribution of DS across households and describe the average annual purchases of processed food across SES groups. We then compared prevalence and quantity of annual purchases across the regions with a Chi^2 test. Second, we used K-mean partition cluster analysis to group the sampled households into clusters based on similarity of their processed food purchases, allowing identification of distinct and predominant patterns in the data. Clustering was done for both 2013 and 2016 separately to analyse temporality of purchase patterns. K-mean partition uses Euclidean distances between observations to empirically estimate clusters within the dataset[28]. Partition clustering is an iterative process that minimizes within-cluster variability while maximizing between-cluster variability at the same time. The technique assigns observations into a pre-defined number of non-overlapping clusters. Each observation is assigned to the cluster with the closest mean. New cluster means are then calculated after each observation is assigned. The process continues iteratively until no observations change clusters [29]. We chose K-means analysis as it is conceptually simple and computationally efficient. Other approaches, e.g. LASSO are available but would offer meaningful advantages if dimensionality in the data were larger [30]. As the number of variables in this analysis is limited there are therefore no apparent gains from using LASSO.

To run the cluster analysis, we calculated the quantity of foods purchased per household member in each food group by dividing household purchase quantity with household size. Clustering was then conducted for 3 to 8 partitions for each year separately. Once the clusters were constructed, boxplots with confidence intervals were created for each food group by clusters to analyse purchase patterns and determine the best fitting number of clusters. Calinski and Harabasz pseudo-F index was used to identify the appropriate number of clusters which is considered as one of the best rules to apply for

For peer review only - http://bmjopen.bmj.com/site/about/guidelines.xhtml

this purpose [31]. It was estimated through a function of ([B](g-1))/([W](N-g)), where B is the between-cluster sum of squares and cross-products matrix, W is the within-cluster sum of squares and cross-products matrix, g is the number of cluster groups and N stands for number of observations[32]. The larger the value of pseudo-F the more clearly defined the cluster structures, and vice versa.

2.3.2. Regression analysis

In the final step, pooled ordinary least square (OLS) regression was used to understand the socio-economic determinants of UPF purchases. We used the logarithm of per household member purchase quantity of UPF as the outcome variable and the following explanatory variables: SES, state and town size of residence dummies, household size, binary variables describing household composition of children across ages (under 1 year, 2-4 years, 5-9 years, 10-14 years and 15-17 years) and binary variables describing durable assets owned by the household, including colour TV, refrigerator, washing machine, laptop/ personal computer, four wheeler, air conditioner. We include time fixed effects in the pooled OLS model to control for macroeconomic changes over the data period. All estimations used robust standard errors (SEs) with clustering at state and town population level. This choice was informed by descriptive and cluster analysis where these spatial descriptors showed relevance in differentiating dietary preferences.

As the SES variable was constructed using education level and ownership of a certain number of durables, we also checked for multicollinearity with binary variables indicating ownership of durables using Variance Inflation Factor (VIF) test. VIF for SES and durables were less than 10 suggesting no issues with multicollinearity. Additionally, we ran regression models together and separately with the SES and durable variables to check if coefficients varied. Our results were robust to these alternative specifications.

2.3.3. Patient and Public Involvement

There are no patients or public participation in this study.

1. RESULTS

1.1. Diversity of processed food purchases

In 2016, three quarters of the households (76%) purchased ten food groups or more out of the 15 used in the study, implying relatively high variety of processed food likely to be consumed by urban Indian households (Supplementary figure 1). Less than 1% of the households purchased all 15 food groups and less than 6% of the households bought seven food groups or less. DS remained constant for 29% of the households, increased for 33% and declined for 37% of the households between 2013 and 2016. Most households purchased less than 10 kgs of UPF per household member. Only 733 households out of 58,878 (1.3%) did not purchase any UPF in 2016 (Supplementary figure 2). The average purchase quantity of UPF in 2016 was 6.4kg.

Figure 1 presents the kernel density curve for annual per household member UPF purchased in kilogram (kg) by SES. It shows that upper and lower class households have more probability weight at low UPF consumption levels compared to middle and upper-middle class households. That is, middle and upper middle class households were more likely to purchase higher quantities of UPF than lower and higher class households. The same kernel density curve for all PF found that households with higher SES were likely to purchase higher quantities of PF (supplementary figure 3).

Figure 1 here

For peer review only - http://bmjopen.bmj.com/site/about/guidelines.xhtml

1.2. Regional variation in processed food purchase

Table 1 presents the annual purchase quantities per household member by food group and zones in 2016 and the colours indicate direction of changes between 2013 and 2016. There is considerable variation in purchase patterns across zones. Overall, the purchases were highest in the North zone (218kg annually per household member), followed by West (153kg), East (127kg) and South (108kg). While UPF purchases made up only small share of these, there was an increase in the purchase of UPF overall (by 0.36kg or ~6% p<0.001) and in East (by 1.2kg or ~21% p<0,001) and West (by 0.39kg or ~9% p<0.001) zones.

Table 1: Annual Purchase Quantity (gm) of Individual Food Groups per Household Member by Zone and Change from 2013 to 2016

**2016**	**Per Capita Consumption by Food Group**				
**Zone**	**North**	**East**	**West**	**South**	**Total**
			**Processed Foods**		
Staples	65,054 (-364)	39,931 (700)	43,936 (-129)	10,597 (496)***	38,663 (158)
Milk	122,516 (-2,780)**	59,872 (4,923)***	83,980 (2,226)***	74,236 (-2,637)***	84,689 (394)
Oils	12,848 (-232)*	12,234 (1,277)***	14,600 (154)	10,818 (328)***	12,723 (348)**
Salt	3,243 (-54)*	4,215 (178)***	3,253 (-138)***	4,158 (-114)***	3,691 (-49)***
Processed Wheat	3,058 (-390)***	1,970 (-64)	867 (-133)***	824 (-431)***	1,550 (-254)***
Tea/Coffee	1,052 (-86)***	913 (41)***	1,082 (9)	1,026 (26)**	1,026 (0)
Spices	1,007 (15)	741 (99)***	460 (40)***	593 (104)***	671 (65)***
Butters	241 (0)	103 (-18)***	106 (16)***	79 (-22)***	127 (-5)*
**Total PF Purchases**	**209,020 (-3889)****	**119,979 (7,136)****	**148,284 (2,045)***	**102,330 (-2,251)****	**143,139 (655)**
			**Ultra-Processed Foods**		
Salty Snack	1,161 (-10)	833 (184)***	1,296 (84)***	623 (-94)***	991 (36)***
Drinks	4,022 (-116)	1,334 (230)***	856 (-43)	1,600 (-311)***	1,833 (-75)**
Ready to Eat Foods	455 (56)***	425 (84)***	334 (42)***	1,526 (315)***	700 (128)***
Sweet Snacks	2,588 (116)***	3,099 (704)***	2,184 (297)***	1,713 (144)***	2,331 (301)***
Milk Drinks	105 (-28)***	845 (-71)***	91 (-18)***	431 (-87)***	339 (-50)***
Breakfast Cereals	311 (63)***	125 (33)***	101 (24)***	199 (-19)**	177 (22)***
Frozen Foods	33 (-6)**	12 (1)	15 (6)***	6 (-5)***	16 (-1)
**Total UPF purchased**	**8,675 (75)**	**6,674 (1,166)*****	**4,876 (390)*****	**6,098 (-58)**	**6,387 (361)*****
**Total PF + UPF Purchase**	**217,695 (-3,814)****	**126,653 (8,302)*****	**153,160 (2,435)****	**108,428 (-2,309)****	**149,526 (1,016)**
**Diversity Score**	**12 *****	**11**	**10**	**11 *****	**11 *****

For peer review only - http://bmjopen.bmj.com/site/about/guidelines.xhtml

Figures in parenthesis show average changes between 2013 and 2016 in grams. *** p-value <0.001, ** p-value <0.01, * p-value <0.05, Cell colour: Green – increase in value, red – decline in value, grey – no change. Beverages were converted from litres to kilograms by multiplying with 1.03.

Across the food groups, most notably, the per household member purchase of ready to eat foods and sweet snacks has increased across urban India by 128gm (~22%, p<0.001) and 301gm (~15%, p<0.001) respectively. Purchase of breakfast foods including sugary cereals were very low (0.18kg on average) but also increased considerably (~14%, p<0.001). While salt purchase declined marginally from 2013 to 2016, it remains twice as high (3.69 kg per year or 10.1gm per day) compared to recommended 5gm per day [33]. Similarly, oil purchase was high with more than 12.3L purchased per household member per year. Purchase of drinks and milk drinks showed an overall decline by 4% and 13% respectively, although an increase in drinks was seen in the East zone (by 0.223L ~17%).

Quantity of purchases per household member declined most in the North zone, driven by reduction in non-UPF purchases. Regardless, the purchase volume in most foods groups remained highest in the North zone compared to other zones. For example, drinks purchases average to 4.02kg in the North zone, which is more than double of the urban average of 1.83kg. Exceptions were ready-to-eat food purchases which were much higher in the South (1.53kg) followed by North (0.46kg) zone. Other exceptions include milk drinks and sweet snacks that are purchased in greater quantity in the East zone (0.82L and 3.1kg respectively). Per household member purchase of frozen foods was the lowest of all food groups (0.016kg on average).

Finally, North zone also had the highest average DS in 2016 (12/15) which had increased since 2013 (p<0.001). This was followed by South and East zone (11/15). West zone had the lowest DS on average (10/15). South zone was the only zone where the decline in average DS was statistically significant (p<0.001).

1.3. Purchase Patterns: Cluster Analysis

We ran cluster analysis by year with number of clusters from 3 to 8. The Calinski and Harabasz pseudo-F index (Supplementary table 2 presents index values for each cluster by year) suggested that three clusters were the optimal partitioning for both years. This was further confirmed by visual inspection of cluster purchase patterns in box plots[2]. Reviewing the purchasing patterns in the three partition model, we found that households fell into distinct clusters that were best characterised by purchase quantities rather than purchases of distinct food categories: low purchase, medium purchase and high purchase of processed foods. This clustering patterns was consistent in 2013 and 2016 data.

Means for key variables of interest by clusters by year are presented in **Error! Reference source not found.**. From 2013 to 2016, the share of households in the low cluster declined from 64% to 54% while the proportion of households in medium and high clusters increased from 32% to 36% and from 4% to 10% respectively. Across the years, the quantities purchased were always smallest for low cluster, followed by medium and high cluster, suggesting that clustering patterns did not change over the four-year period. Between 2013 and 2016 overall PF purchase volumes declined in all three clusters, while UPF purchases increased for low (p<0.01) and high purchase cluster. Purchases of PF and UPF were more than three times greater in high cluster compared to low cluster. Sweet snacks and ready-to-eat foods were two categories that had consistent increase in all three clusters while milk and milk drinks showed a consistent decrease. Although, low purchase cluster bought less of UPF, the share of UPF in

[2] Due to large number of graphs, these are not presented in the paper but are available upon request.

For peer review only - http://bmjopen.bmj.com/site/about/guidelines.xhtml

their average share of food basket was higher (5.2% in 2016) than medium (3.8%) and high (3.9%) purchase clusters[3]. The difference between cluster means was statistically significant (p<0.001).

The high purchase cluster households were likely to have smaller household size than low and medium purchase clusters (Table 2). In line with this, households in low purchase cluster were more likely to have children in every age category compared to households in middle and high purchase clusters. In term of geographical distribution, households in high purchase cluster were more likely to be from North zone than other zones (58% in 2013 and 49% in 2016). The medium purchase cluster included relatively similar share of households from North and West (38% and 32% respectively in 2013) which remained relatively consistent in 2016. Households from the biggest towns (> 1 million in population) were more likely to be in the high purchase cluster, which may be due to a larger availability of processed food in bigger towns and cities.

Table 2: Processed food purchase patterns for urban India for 2013 and 2016

	2013			2016		
**Purchase Clusters**	**Low**	**Medium**	**High**	**Low**	**Medium**	**High**
Number (%) of HHs	37,331 (63.4%)	19,059 (32.4%)	2,488 (4.2%)	31,883 (54.1%)	21,422 (36.4%)	5,573 (9.5%)
Dietary diversity	10.7	11.1	11.1	10.6	11.0	11.2
Household size	5.1	4.1	2.7	5.0	4.2	2.9
**Average Annual Per Capita Purchase of Food Groups in gm**						
Milk	47,345	129,956	288,926	41,698	111,331	228,233
Staples	28,733	50,734	91,439	24,848	49,069	77,697
Oils	10,913	14,038	21,577	10,725	14,058	19,019
Salt	3,518	3,885	5,953	3,332	3,824	5,235
Processed Wheat	1,309	2,506	3,867	1,060	1,878	3,094
Tea/Coffee	843	1,243	2,115	811	1,168	1,709
Spices	522	687	1,266	572	714	1,069
Butters & Cheese	64	226	426	54	165	402
**Total PF Purchase**	**93,246**	**203,276**	**415,569**	**83,100**	**182,206**	**336,458**
Drinks	918	3,248	6,488	825	2,287	5,849
Sweet Snacks	1,807	2,306	3,266	2,061	2,396	3,623
Salty Snacks	713	1,325	1,752	683	1,208	1,925
Ready to Eat Foods	484	698	930	595	704	1,285
Milk Drinks	361	422	556	320	328	495
Breakfast Cereals	86	246	494	85	228	513
Frozen Food	5	33	69	5	21	55
**Total UPF Purchase**	**4,375**	**8,278**	**13,554**	**4,573**	**7,172**	**13,745**
**% of UPF in Total Purchase**	**4.48%**	**3.91%**	**3.16%**	**5.22%**	**3.79%**	**3.92%**
**Total PF + UPF Purchase**	**97,621**	**211,554**	**429,123**	**87,673**	**189,378**	**350,202**
**Percentage of Households with a Child in Age Group**						
Infant	3%	2%	0%	6%	4%	2%
<1 year	5%	3%	1%	3%	2%	1%
2-4 years	15%	10%	4%	14%	10%	4%
5-9 years	26%	18%	7%	23%	16%	8%
10-14 years	34%	25%	11%	29%	21%	10%

[3] We conducted ANOVA (p-value<0.001) and multivariate test of means (p-value<0.001) to test statistical significance in the difference in UPF purchases by clusters for each year. Null hypothesis of equal means was rejected confirming the difference was significant between clusters.

For peer review only - http://bmjopen.bmj.com/site/about/guidelines.xhtml

15-17 years	26%	19%	8%	23%	18%	9%
**Zone**						
North	11%	38%	58%	9%	33%	49%
East	26%	12%	7%	26%	14%	12%
West	31%	32%	21%	30%	34%	26%
South	32%	18%	14%	34%	20%	13%
**Town size**						
40 Lakhs +	26%	25%	18%	27%	23%	23%
10-40 Lakhs	31%	44%	53%	30%	43%	47%
05-10 Lakhs	15%	11%	7%	15%	11%	9%
01-05 Lakhs	14%	11%	14%	15%	11%	11%
<= 01 Lakhs	14%	10%	8%	14%	10%	10%
**Socio-Economic Status (SES)**						
Lower Class	13%	4%	5%	14%	5%	4%
Middle Class	29%	14%	13%	30%	18%	10%
Upper Middle Class	31%	26%	26%	31%	29%	22%
Upper Class	27%	56%	56%	25%	49%	64%
**Durables/Assets**						
Two Wheeler	53%	70%	66%	58%	70%	69%
Refrigerator	53%	80%	80%	58%	78%	84%
Washing Machine	21%	50%	55%	28%	53%	66%
Four Wheeler	19%	38%	36%	23%	35%	40%
Laptop/Personal Computer	10%	24%	29%	16%	27%	38%
Air Conditioner	5%	16%	24%	6%	17%	34%

As expected, the medium and high purchase clusters contained a greater proportion of households from upper middle and upper class. 56% of households purchasing high quantities of processed food were from upper class in 2013 which increased to 64% in 2016. Furthermore, households purchasing medium and high level of all PF (including UPF) were more likely to own durables such as refrigerators, washing machine, four wheeler, laptop and air conditioner.

Figure 2 presents the share of individual PF (Panel A) and UPF (Panel B) food groups as percentage of total PF and UPF purchases respectively. Panel A shows that the low cluster had a greater share of the foods consumed on a daily basis, such as staples, oils, tea/coffee and spices. For this cluster almost half of the UPF purchases (45%) were sweet snacks (Panel B). This cluster also had the highest share of ready-to-eat foods (13% of UPF) and milk drinks (7% of UPF). In comparison, the high cluster purchased a larger proportion of milk (68% of PF). They also had a higher share of drinks (43% of UPF), breakfast cereals (4% of UPF) and frozen foods (0.4% of UPF). Medium purchase cluster stood out for slightly greater share of salty snacks purchases in comparison to low and high purchase clusters.

Figure 2 here

Finally, we estimated multinomial logit models for groups identified by cluster analysis. The results are presented in supplementary table 3. These results confirmed that higher SES, having older children and durables to be positively and significantly associated with medium and high clusters purchasing higher quantities of all PF than the low cluster.

For peer review only - http://bmjopen.bmj.com/site/about/guidelines.xhtml

1.4. Determinants of Ultra- Processed Food Purchase: Regression analysis

Error! Reference source not found. presents the pooled OLS model for UPF quantity per household member purchased in 2013 and 2016. We found that SES, large town sizes, having children under the age 14 in the household, and ownership of durable assets were positively and significantly related to UPF purchase. Of particular importance is the SES. An upper class household purchased 34% ($p<0.001$) ($Y= e^{0.29} = 1.3364$ or 34%) more UPF than a lower class household. Both middle and upper-middle class households purchased more UPF than lower class household by 22% ($p<0.001$) and 14% ($p<0.001$), respectively. Households from towns with more than 4m inhabitants purchased 50% ($p<0.01$) more UPF than households from the smallest towns (population of less than 100,000). In comparison, households in the towns with 1-4m and 0.51m inhabitants purchased 39% ($p<0.01$) and 35% ($p<0.05$) more UPF to households from the smallest towns, respectively. An additional household member was associated with a 13% ($p<0.001$) less UPF purchased per member. Having children under the age of 14 years had a positive (6-9%) and significant association ($p<0.05$) with UPF purchase.

For peer review only

Table 3: Multivariate analysis of UPF purchase quantity (g) per household member (UPF qphm)

Outcome variable: log(UPF qphm) // Independent Variables	Coefficient	Standard Error	p-value	95% CI	
Base - SES - Lower Class					
SES- Middle Class	0.127	0.032	<0.001	0.062	0.192
SES- Upper Middle Class	0.196	0.043	<0.001	0.109	0.282
SES- Upper Class	0.290	0.049	<0.001	0.193	0.387
Base - Town population <100k					
Town Population - 500k>=X>100k	0.154	0.133	0.252	-0.112	0.421
Town Population - 1mil>=X>500k	0.300	0.129	0.023	0.042	0.557
Town Population - 4mil>=X>1mil	0.331	0.119	0.007	0.094	0.568
Town Population - >4mil	0.402	0.143	0.007	0.117	0.688
Household Size	-0.143	0.005	<0.001	-0.154	-0.132
Infant	0.067	0.028	0.020	0.011	0.122
Children under 1 year	0.080	0.027	0.004	0.027	0.133
Children 2-4 years	0.086	0.011	<0.001	0.065	0.108
Children 5-9 years	0.088	0.013	<0.001	0.062	0.115
Children 10-14 years	0.058	0.010	<0.001	0.038	0.077
Children 15-17 years	0.008	0.011	0.430	-0.013	0.029
Durable: Colour TV	0.183	0.040	<0.001	0.103	0.264
Durable: Refrigerator	0.090	0.024	<0.001	0.043	0.138
Durable: Washing Machine	0.124	0.031	<0.001	0.062	0.186
Durable: Laptop/PC	0.194	0.025	<0.001	0.145	0.243
Durable: Four Wheeler	0.164	0.037	<0.001	0.089	0.239
Durable: Air Conditioner	0.352	0.045	<0.001	0.261	0.443
Time Effect (base 2013)	0.016	0.027	0.563	-0.039	0.070
Constant	1.422	0.152	<0.001	1.119	1.725
Observations	58,878				
State Effect	Yes				

Notes: Pooled OLS with robust standard errors clustered at state and town population level; *** $p<0.001$, ** $p<0.01$, * $p<0.05$

Ownership of durables had a positive and statistically significant association with quantity of UPF purchased. Households that owned an air conditioner purchased 42% ($p<0.001$) more UPF compared to those that did not. Households who owned a computer (laptop or PC) or colour TV were associated

with 20-21% (p<0.001) more UPF purchase than those households that did not own them (note that only 22% of households owned laptop/PCs but 98% owned a colour TV in 2016). Ownership of a four wheeler and washing machine increased purchase by 18% (p<0.001) and 13% (p<0.001) respectively, while ownership of a refrigerator only increased purchase by 9%. About a third (37%) of the households did not own a refrigerator in 2016 suggesting that households might be primarily purchasing UPF that do not require cold storage.

2. DISCUSSION

This paper used a unique dataset for urban Indian household purchases to assess patterns and socio-economic determinants of processed, and ultra-processed food purchases in 2013 and 2016. We found that three quarters of urban Indian households purchase a higher variety of processed food (10 out of 15 food groups). However, 60% of them purchased processed food at small quantities that was less than 150kgs per household member annually of which vast majority were commonly consumed products such as milk, oil, atta, rice and salt.

This analysis, to our knowledge, is first to use household purchase data to examine purchases of UPF in urban India and we found that the average annual purchase of these foods was relatively low at 6.4kg per household member, but importantly it had increased by 6% between 2013 and 2016. In comparison, for example, in the US, share of calories from UPF consumption increased by 7.5% between a 16-year time period from 2002 and 2018 [34]. We also found significant regional variation in UPF purchase, as North Indian households purchase on average 8.7kg UPF per household member annually. This was followed by East (6.7kg), South (6.1kg) and West (4.9kg) zones. However increases in UPF purchases were most notable in East (21%) and West (9%). Northern households also purchased higher quantity of all processed foods. On average they purchased 217kg of processed food per household member in comparison to the national average of 150kg. Southern households purchased less than half of northern average (108kg), even though the region has high levels of urbanisation. Higher urbanisation levels in South India may not translate to high consumption of PF and UPF because of the regional food cultures and regional heterogeneity in preferences in food consumption. Our analysis confirms that regionality determines purchase patterns of PF and UPF, as suggested by previous dietary studies on India [20, 35]. A clear health risk is the high average level of annual salt purchases that was at over 3.7kg per person which is nearly twice as high as WHO guideline of 1.8 kg per year[36]. Both quantity and preferences for food groups varied across urban regions in the country.

Cluster analysis found three distinct purchase clusters amongst urban Indian consumers - low (54% of households in 2016), medium (36% of households) and high (10% of households) purchase clusters in 2016. Despite the short period of time between two data points we saw a shift towards medium and high purchase clusters in 2016 with greater share of households accounted by these two clusters compared to 2013. Medium and high purchase clusters were more likely to have households from higher SES and living in big cities. Households in high purchase cluster bought more of every type of food groups, including three times more of UPF than low cluster. However the low purchase cluster had the greatest volume share of more commonly consumed processed food such as rice, atta, oil, tea, coffee, and spices. High purchase cluster had a higher share of higher value processed food such as milk, butter & cheese, drinks, breakfast cereals and frozen foods. Overall purchases of UPF were relatively low, even in the high purchase cluster (e.g. 5.7L of drinks, 3.6kg of sweet snacks and 1.9kg of salty snacks per year) but it was increasing both in high and low purchase clusters. Sweet snacks and ready-to-eat foods in particular showed greatest increase between 2013 and 2016 with both being the two most prominent UPF purchased by low purchase cluster. Finally, we found that quantity of

For peer review only - http://bmjopen.bmj.com/site/about/guidelines.xhtml

UPF purchased was positively associated with socioeconomic status, town size, and presence of children younger than fourteen as well as ownership of durables, such as refrigerator and a four wheeler vehicle.

Our results are consistent with the limited literature available in this area. For example, Baker and colleagues [7] who used Euromonitor International Passport database to estimate sales of ultra-processed food and beverages found that in 2019, per capita sales of processed food stood at less than 50 kgs in India, while the compounding annual growth rate for sales between 2009-2019 was 6%. Regional differences for dietary patterns were also observed by other studies of Indian diets [13, 20, 35]. For example, using NSSO food purchase data for 2011-12, Western Indian households were found to have lower dietary diversity score than rest of the country [20]. Our analysis also found diversity of processed food purchased to be one group lower for households in Western zone, on average. A recent study based on 24hour recall data for North and South Indian households found salt intake to be 11gms per day or 4kgs annually, similarly to our findings [37]. The study also found that salt intake was mainly from added salt during cooking. Our results confirm that in comparison to Latin American countries, purchase of sugar-sweetened beverages in India is relatively low [38].

The positive association between processed food consumption and socioeconomic position however has been observed in studies conducted in other developing countries [39-43]. In Mexico for example, high socio-economic status individuals were found to have 3.4-7.8 percent point greater share of energy contribution of UPF in the diet and the study also found regional differences in UPF consumption. In Brazil, similarly, the contribution of UPF to energy in the diet has been found to be 20% less among the lowest income group compare to the highest. To the contrary, the evidence in developed countries (e.g. the US [44], Canada [39], Australia [45], Portugal[46]) seems to indicate that UPF consumption is higher among lower socio-economic status individuals or households. Moubarac et al. hypothesise that this is due to UPF products costing relatively more in developing countries [39] whereas Magalhaes et al. point that as countries become wealthier its growing middle classes may be consuming more UPF to exhibit socio-economic status [39, 46]. While we were able to provide new insight into dietary behaviours in urban India over time and by population groups combining cluster analysis with multivariate analysis to investigate determinants of UPF purchases, our work has limitations. First, the dataset did not include unprocessed food purchases, which would allow a more refined analysis of dietary transitions towards processed and ultra-processed foods. Despite this, the analysis of processed and ultra-processed food purchases is relevant on its own, particularly as the NSSO has thus far lacked detail on these foods. In future should new NSSO data still lack detail on processed food a useful avenue for research could be a matching analysis of NSSO and Kantar data. . Law, Green [13] found that for most food items which could be compared across the two datasets, such cold beverages, milk (in liquid form) and edible oils, the difference between NSSO data and Kantar were small [13].

Secondly, also inevitable due to lack of data, is the missing information of out-of-home purchases which include purchases made on-the-go as well as food consumed in restaurants, cafes or work and study places. Additionally, we do not know the exact composition of the household in terms of age and gender and therefore have to assume that food and beverage purchases are shared equally across household members. Finally, Kantar data is based on purchase of food items rather than actual consumption. These limitations however, are not unique to these data. Research using similar granular purchase data in high-income countries also make this assumption[47]. Regardless of the limitations, our analysis provides new insight into diets in India and helps to improve existing evidence on the nature of processed and ultra-processed food in urban Indian diets, which are often linked to increasing obesity and diet-related non-communicable diseases.

For peer review only - http://bmjopen.bmj.com/site/about/guidelines.xhtml

Our findings underline important differences and changes in dietary patterns over time in urban Indian population. The results have critical implications for ongoing debates on the role of processed and ultra-processed food in low and middle income countries. Key concerns are low but rising purchase of UPF, persistently high levels of salt purchase and growing trends towards sweet and salty snacks, breakfast cereals and ready-to-eat foods across the country but particularly in Northern India. Significant role of socio-economic status, town size and regional preferences suggest the need for tailored regional and city level interventions to curb the low but growing purchase of UPF. The results are concerning given the links between lifestyle changes and speed of urbanisation [48] especially in tier-2 and 3 cities of the country along with the recently released NFHS survey results for 2019-20 that found a dramatic rise in obesity among children under five in 20 out of 22 Indian states [49].

For peer review only

For peer review only - http://bmjopen.bmj.com/site/about/guidelines.xhtml

3. Data availability statement

Kantar data is proprietary data and thus cannot be made publicly available. Requests regarding data availability should be made to Kantar Worldpanel Division India.

4. Ethics statement

Ethics approval was not required as the study uses secondary anonymised data

5. Competing Interests

None declared.

6. Funding

This study forms part of the Sustainable and Healthy Food Systems (SHEFS) programme supported by the Wellcome Trust's Our Planet, Our Health programme [grant number: 205200/Z/16/Z]. LC is funded by a UK Medical Research Council fellowship (grant number MR/P021999/1).

7. Contributorship Statement

MT, LC, CL designed the study. MT conducted the data analysis. MT, LC, CL, RG and BS interpreted the data and the analysis findings. MT wrote the first draft. CL, LC, BS and RG critically revised and edited the manuscript for intellectual content. All authors have read and approved the final draft.

For peer review only

References

1. ICMR and PHFI, *Health of the Nation's States—The India State-Level Disease Burden Initiative. New Dehli: Indian Council of Medical Research.* 2017, Institute for Health Metrics and Evaluation, Public Health Foundation of India.
2. Economic Times, *Unhealthy diet, lack of physical activities lead to drastic rise in obesity among adults and kids Men and women with BMI over or equal to 25.0 kg/m2 were counted as obese*, in *Economic Times*. 2020: New Delhi.
3. IIPS and ICF, *National Family Health Survey (NFHS-4), 2015-16*. 2017, International Institute for Population Sciences India. Mumbai.
4. Popkin, B.M., L.S. Adair, and S.W. Ng, *Global nutrition transition and the pandemic of obesity in developing countries.* Nutr Rev, 2012. **70**(1): p. 3-21.
5. Garnett, T., *Plating up solutions.* 2016. **353**(6305): p. 1202-1204.
6. Bren d'Amour, C., et al., *Urbanization, processed foods, and eating out in India.* Global Food Security, 2020. **25**: p. 100361.
7. Baker, P., et al., *Ultra-processed foods and the nutrition transition: Global, regional and national trends, food systems transformations and political economy drivers.* Obesity Reviews, 2020. **21**(12): p. e13126.
8. Moodie, R., et al., *Ultra-Processed Profits: The Political Economy of Countering the Global Spread of Ultra-Processed Foods – A Synthesis Review on the Market and Political Practices of Transnational Food Corporations and Strategic Public Health Responses.* International Journal of Health Policy and Management, 2021.
9. Mendonça, R.D., et al., *Ultra-Processed Food Consumption and the Incidence of Hypertension in a Mediterranean Cohort: The Seguimiento Universidad de Navarra Project.* Am J Hypertens, 2017. **30**(4): p. 358-366.
10. Mendonça, R.D., et al., *Ultraprocessed food consumption and risk of overweight and obesity: the University of Navarra Follow-Up (SUN) cohort study.* Am J Clin Nutr, 2016. **104**(5): p. 1433-1440.
11. Srour, B., et al., *Ultraprocessed Food Consumption and Risk of Type 2 Diabetes Among Participants of the NutriNet-Santé Prospective Cohort.* JAMA Intern Med, 2020. **180**(2): p. 283-291.
12. Green, R., et al., *Dietary patterns in India: a systematic review.* Br J Nutr, 2016. **116**(1): p. 142-8.
13. Law, C., et al., *Purchase trends of processed foods and beverages in urban India.* Glob Food Sec, 2019. **23**: p. 191-204.
14. Sharma, M., et al., *A comparison of the Indian diet with the EAT-Lancet reference diet.* BMC Public Health, 2020. **20**(1): p. 812.
15. Euromonitor, *Passport Global Market Information Database*, E. International, Editor. 2020: London.
16. Daniel, C.R., et al., *A cross-sectional investigation of regional patterns of diet and cardio-metabolic risk in India.* Nutr J, 2011. **10**: p. 12.
17. Satija, A., et al., *Dietary patterns in India and their association with obesity and central obesity.* Public Health Nutr, 2015. **18**(16): p. 3031-41.
18. Roy, D., et al., *Snack Food Consumption across the Pune Transect in India: A Comparison of Dietary Behaviors Based on Consumer Characteristics and Locations.* 2021. **13**(12): p. 4325.
19. Kantar, *Worldpanel Division, India*, Kantar, Editor. 2018.
20. Tak, M., B. Shankar, and S. Kadiyala, *Dietary Transition in India: Temporal and Regional Trends, 1993 to 2012.* Food and Nutrition Bulletin, 2019. **40**(2): p. 254-270.
21. Aguilar, A., E. Gutierrez, and E. Seira, *The effectiveness of sin food taxes: Evidence from Mexico.* J Health Econ, 2021. **77**: p. 102455.

For peer review only - http://bmjopen.bmj.com/site/about/guidelines.xhtml

22. Taillie, L.S., et al., *Changes in food purchases after the Chilean policies on food labelling, marketing, and sales in schools: a before and after study.* The Lancet. Planetary health, 2021. **5**(8): p. e526-e533.
23. Stacey, N., et al., *Changes in beverage purchases following the announcement and implementation of South Africa's Health Promotion Levy: an observational study.* Lancet Planet Health, 2021. **5**(4): p. e200-e208.
24. Smith, R.D., et al., *Are sweet snacks more sensitive to price increases than sugar-sweetened beverages: analysis of British food purchase data.* 2018. **8**(4): p. e019788.
25. Yau, A., et al., *Changes in household food and drink purchases following restrictions on the advertisement of high fat, salt, and sugar products across the Transport for London network: A controlled interrupted time series analysis.* PLOS Medicine, 2022. **19**(2): p. e1003915.
26. Bandy, L., et al., *The use of commercial food purchase data for public health nutrition research: A systematic review.* PLOS ONE, 2019. **14**(1): p. e0210192.
27. Monteiro, C.A., et al., *Ultra-processed foods: what they are and how to identify them.* Public Health Nutrition, 2019. **22**(5): p. 936-941.
28. Jacobson, H.N. and J.L. Stanton, *Pattern analysis in nutrition.* Clinical nutrition, 1986. **5**: p. 249–253.
29. Aldenderfer, M.S. and R.K. Blashfield, *Cluster Analysis. Quantitative Applications in the Social Sciences*. 1984, Newbury Park, CA: Sage Publications.
30. Narayanan, L., A.S. Babu, and M.R. Kaimal. *Projected Clustering with LASSO for High Dimensional Data Analysis*. 2015. Cham: Springer International Publishing.
31. Milligan, G.W. and M.C. Cooper, *An examination of procedures for determining the number of clusters in a data set.* Psychometrika, 1985. **50**(2): p. 159-179.
32. Caliński, T. and J. Harabasz, *A dendrite method for cluster analysis.* Communications in Statistics, 1974. **3**(1): p. 1-27.
33. WHO, *Salt reduction*, in *Factsheets*. 2020, WHO.
34. Juul, F., et al., *Ultra-processed food consumption among US adults from 2001 to 2018.* The American Journal of Clinical Nutrition, 2021. **115**(1): p. 211-221.
35. Choudhury, S., et al., *What underlies inadequate and unequal fruit and vegetable consumption in India? An exploratory analysis.* Global Food Security, 2020. **24**: p. 100332.
36. WHO, *Report of the Formal Meeting of Member States to conclude the work on the comprehensive global monitoring framework, including indicators, and a set of voluntary global targets for the prevention and control of communicable diseases*. 2012.
37. Johnson, C., et al., *Sources of Dietary Salt in North and South India Estimated from 24 Hour Dietary Recall.* Nutrients, 2019. **11**(2): p. 318.
38. Popkin, B.M. and C. Hawkes, *Sweetening of the global diet, particularly beverages: patterns, trends, and policy responses.* The lancet. Diabetes & endocrinology, 2016. **4**(2): p. 174-186.
39. Moubarac, J.C., et al., *Consumption of ultra-processed foods and likely impact on human health. Evidence from Canada.* Public Health Nutr, 2013. **16**(12): p. 2240-8.
40. Djupegot, I.L., et al., *The association between time scarcity, sociodemographic correlates and consumption of ultra-processed foods among parents in Norway: a cross-sectional study.* BMC Public Health, 2017. **17**(1): p. 447.
41. Khandpur, N., et al., *Sociodemographic factors associated with the consumption of ultra-processed foods in Colombia.* Revista de saude publica, 2020. **54**: p. 19-19.
42. Simões, B.D.S., et al., *Consumption of ultra-processed foods and socioeconomic position: a cross-sectional analysis of the Brazilian Longitudinal Study of Adult Health (ELSA-Brasil).* Cad Saude Publica, 2018. **34**(3): p. e00019717.
43. Marrón-Ponce, J.A., et al., *Energy contribution of NOVA food groups and sociodemographic determinants of ultra-processed food consumption in the Mexican population.* Public Health Nutrition, 2018. **21**(1): p. 87-93.

For peer review only - http://bmjopen.bmj.com/site/about/guidelines.xhtml

44. Baraldi, L.G., et al., *Consumption of ultra-processed foods and associated sociodemographic factors in the USA between 2007 and 2012: evidence from a nationally representative cross-sectional study.* 2018. **8**(3): p. e020574.
45. Marchese, L., et al., *Ultra-processed food consumption, socio-demographics and diet quality in Australian adults.* Public Health Nutrition, 2022. **25**(1): p. 94-104.
46. Magalhães, V., et al., *Associated factors to the consumption of ultra-processed foods and its relation with dietary sources in Portugal.* J Nutr Sci, 2021. **10**: p. e89.
47. Scheelbeek, P.F.D., et al., *Potential impact on prevalence of obesity in the UK of a 20% price increase in high sugar snacks: modelling study.* 2019. **366**: p. l4786.
48. Misra, A., et al., *Nutrition transition in India: secular trends in dietary intake and their relationship to diet-related non-communicable diseases.* J Diabetes, 2011. **3**(4): p. 278-92.
49. Sciences, I.I.f.P., *National Family Health Survey (NFHS-5) 2019–2020 fact sheets: Key Indicators 22 States/UTs from phase I*, M.o.H.a.F.W. International Institute of Population Sciences, Government of India. , Editor. 2020.

For peer review only

For peer review only - http://bmjopen.bmj.com/site/about/guidelines.xhtml

8. Figure legend

Figure 1: Kernel Density Curves for UPF Purchase by Socio-Economic Status in 2016

Figure 2: Share of Food Groups Purchase Quantity by Clusters in 2016

For peer review only

For peer review only - http://bmjopen.bmj.com/site/about/guidelines.xhtml

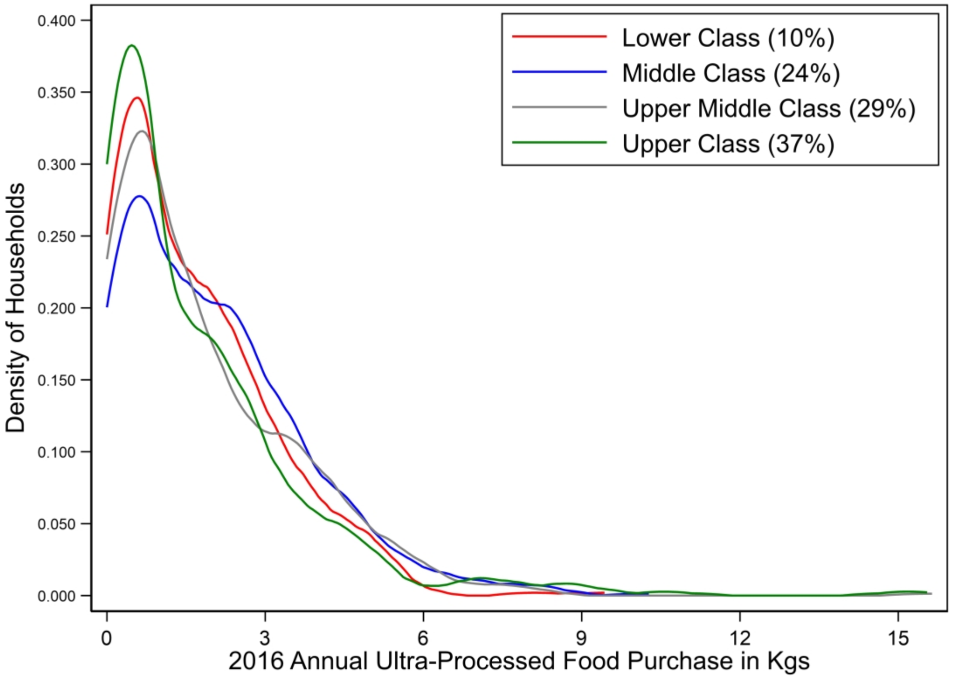

Figure 1: Kernel Density Curves for UPF Purchase by Socio-Economic Status in 2016

2205x1604mm (38 x 38 DPI)

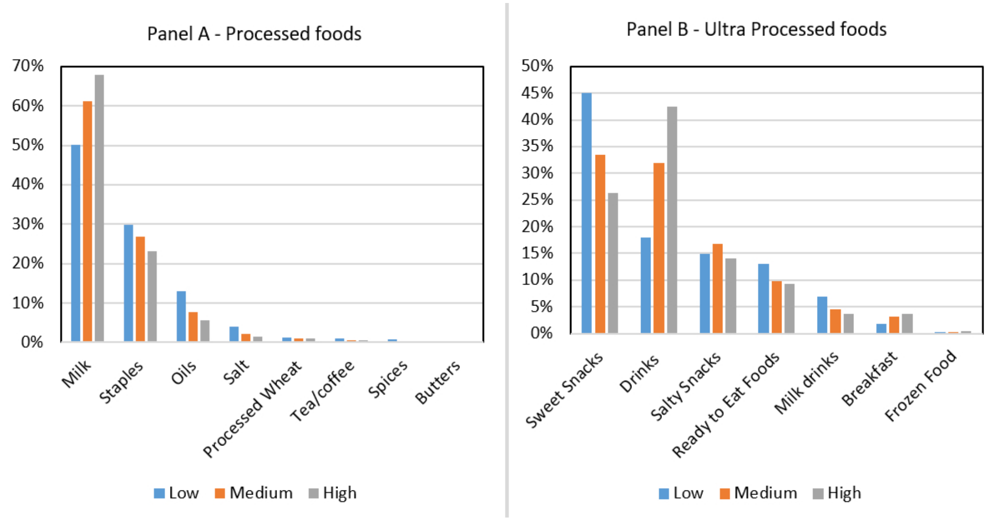

Figure 2: Share of Food Groups Purchase Quantity by Clusters in 2016

734x378mm (38 x 38 DPI)

Supplementary Appendix

Supplementary Table 1: Food Groups

	Food group	Individual food items
1	Staples	Basmati rice [a], atta
2	Milk	Milk
3	Oils	Edible oil, ghee, vanaspati
4	Salt	Salt
5	Processed Wheat	Pasta, bread
6	Tea and Coffee	Tea, coffee
7	Spices	Spices
8	Butters and Cheese	Butter, cheese
9	Salty Snacks (UPF)	Chips, collet, popcorn, potato chips, puffed snacks, traditional snacks
10	Drinks (UPF)	Carbonated drinks, juices, milk based drink, squash
11	Ready to Eat Foods (UPF)	Cooking paste, cook mix, noodle, soup, sauces, pickles, ready meals
12	Sweet Snacks (UPF)	Biscuits, rusk, chocolate, chocolate spread, peanut, butter, jams
13	Milk Drinks (UPF)	Milk food drinks (e.g. malt drinks, chocolate milk), Milk powder (including infant milk powder)
14	Frozen Foods (UPF)	Frozen foods (e.g. fish fingers, samosas)
15	Breakfast cereals (UPF)	Breakfast cereals

Note: [a] Includes data on basmati rice only, which is a higher quality rice and thus does not include the non-basmati variety of rice that is consumed by most Indians

Supplementary Table 2: Calinski and Harabasz pseudo-F index

Clusters	2013	2016
3	37,991.23	41,112.57
4	36,085.22	37,939.35
5	37,141.41	37,687.36
6	35,393.65	36,583.87
7	36,005.66	35,706.64
8	34,889.70	34,865.53

Supplementary Table 3: Cluster multinomial logit regressions

	Cluster	
VARIABLES	2013	2016
Base Cluster Low		
Cluster - Medium		
SES- Middle Class	1.388*** (0.102)	1.548*** (0.097)
SES- Upper Middle Class	2.133*** (0.247)	2.243*** (0.23)
SES- Upper Class	3.526*** (0.531)	3.792*** (0.426)
Town Population - 500k>=X>100k	1.048 (0.161)	0.979 (0.147)
Town Population - 1mil>=X>500k	0.934 (0.159)	1.008 (0.172)
Town Population - 4mil>=X>1mil	1.041 (0.191)	0.973 (0.167)

For peer review only

Town Population - >4mil	0.835 (0.217)	0.756 (0.173)
Household Size	0.542*** (0.017)	0.564*** (0.022)
Infant	1.195* (0.089)	1.089 (0.098)
Children under 1 year	1.001 (0.067)	1.042 (0.099)
Children 2-4 years	1.044 (0.052)	0.996 (0.039)
Children 5-9 years	0.883*** (0.029)	0.898* (0.04)
Children 10-14 years	0.836*** (0.027)	0.837*** (0.023)
Children 15-17 years	0.706*** (0.028)	0.805*** (0.024)
Durable: Colour TV	1.17* (0.086)	1.5*** (0.137)
Durable: Refrigerator	1.534*** (0.074)	1.358*** (0.074)
Durable: Washing Machine	1.45*** (0.1)	1.412*** (0.115)
Durable: Laptop/PC	1.271*** (0.069)	1.171** (0.058)
Durable: Four Wheeler	1.483** (0.179)	1.288** (0.122)
Durable: AC	1.846*** (0.216)	1.816*** (0.201)
Constant	5.826*** (1.307)	7.4*** (1.767)
Cluster - High		
SES- Middle Class	1.546* (0.283)	1.665*** (0.214)
SES- Upper Middle Class	3.278*** (0.691)	3.64*** (0.617)
SES- Upper Class	5.87*** (1.507)	9.556*** (1.95)
Town Population - 500k>=X>100k	1.35 (0.473)	0.808 (0.193)
Town Population - 1mil>=X>500k	0.629 (0.23)	0.718 (0.181)
Town Population - 4mil>=X>1mil	1.143 (0.321)	0.742 (0.201)
Town Population - >4mil	0.892 (0.418)	0.436* (0.181)
Household Size	0.157*** (0.025)	0.174*** (0.018)
Infant	0.923 (0.297)	1.289 (0.229)
Children under 1 year	1.403 (0.373)	0.996 (0.308)
Children 2-4 years	1.2 (0.255)	1.104 (0.168)
Children 5-9 years	0.747** (0.072)	0.981 (0.085)
Children 10-14 years	0.804 (0.103)	0.734** (0.067)
Children 15-17 years	0.486*** (0.054)	0.731*** (0.051)
Durable: Color TV	1.668*** (0.23)	2.714*** (0.476)
Durable: Refrigerator	1.435** (0.17)	1.428** (0.16)
Durable: Washing Machine	1.597** (0.215)	1.681*** (0.194)
Durable: Laptop/PC	1.478** (0.185)	1.335** (0.113)
Durable: Four Wheeler	2.208*** (0.439)	1.916*** (0.26)
Durable: AC	3.199*** (0.844)	3.433*** (0.552)
Constant	15.488*** (7.106)	38.817*** (17.155)
Observations	58,878	58,878
State Effect	Yes	Yes

Notes: Robust standard errors in parentheses; *** p<0.001, ** p<0.01, * p<0.5

For peer review only

Supplementary Figure 1: Distribution of Processed Food Group Diversity Score in 2016

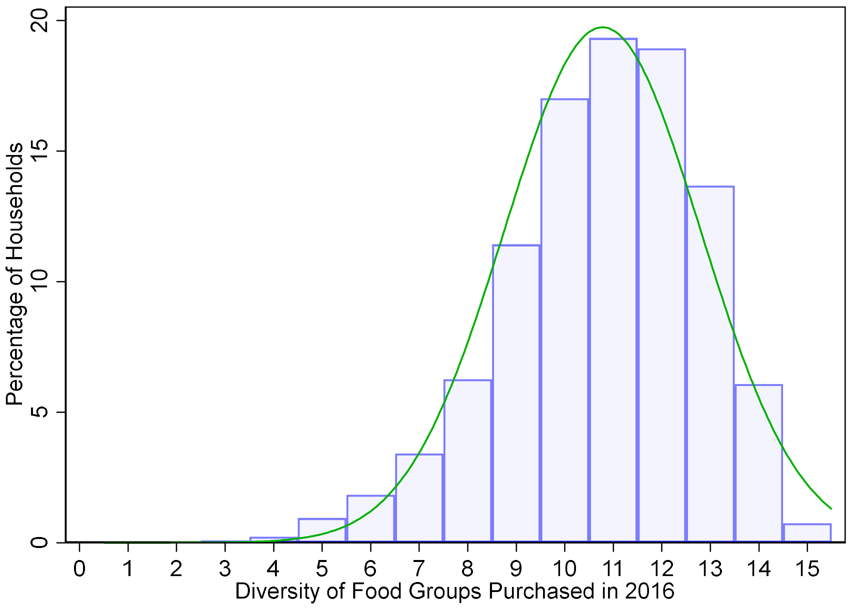

Supplementary Figure 2: Distribution of annual Ultra-Processed food and Beverages Quantity in 2016

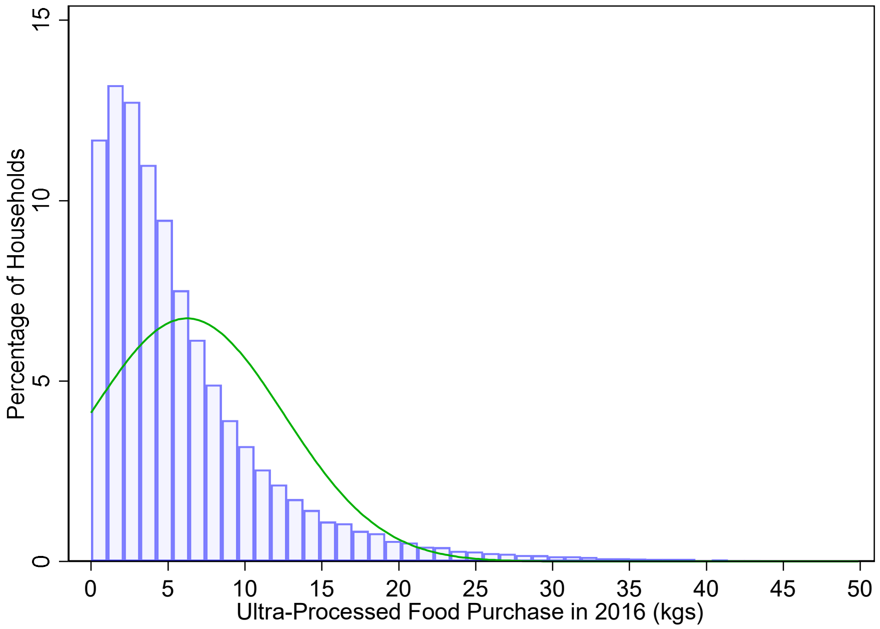

Notes: For readability purposes, the distribution excludes 140 households (0.2%) that purchased more than 50kgs of UPF per household member.

Supplementary Figure 3: Kernel Density Curves for Processed Food Purchase by Socio-Economic Status in 2016

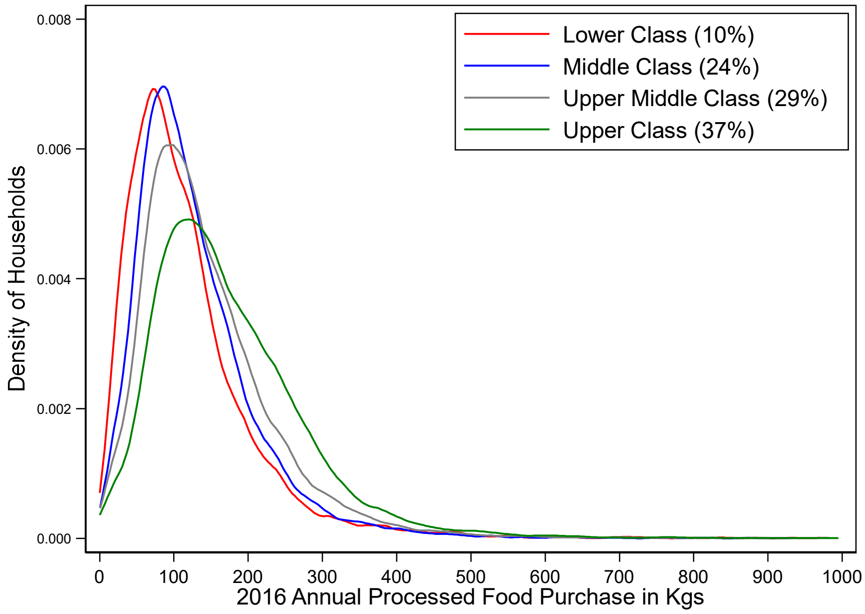

Supplement: Author's manuscript [file bmjopen-2022-062254.draft_revisions.pdf]
